# Does Don Fisher's high-pressure manifold model account for phloem transport and resource partitioning?

**DOI:** 10.3389/fpls.2013.00184

**Published:** 2013-06-19

**Authors:** John W. Patrick

**Affiliations:** School of Environmental and Life Sciences, The University of NewcastleCallaghan, NSW, Australia

**Keywords:** Bulk flow, hydraulic conductance, hydrostatic pressure, plasmodesmata, phloem transport, symplasmic phloem unloading, resource partitioning

## Abstract

The pressure flow model of phloem transport envisaged by Münch (1930) has gained wide acceptance. Recently, however, the model has been questioned on structural and physiological grounds. For instance, sub-structures of sieve elements may reduce their hydraulic conductances to levels that impede flow rates of phloem sap and observed magnitudes of pressure gradients to drive flow along sieve tubes could be inadequate in tall trees. A variant of the Münch pressure flow model, the high-pressure manifold model of phloem transport introduced by Donald Fisher may serve to reconcile at least some of these questions. To this end, key predicted features of the high-pressure manifold model of phloem transport are evaluated against current knowledge of the physiology of phloem transport. These features include: (1) An absence of significant gradients in axial hydrostatic pressure in sieve elements from collection to release phloem accompanied by transport properties of sieve elements that underpin this outcome; (2) Symplasmic pathways of phloem unloading into sink organs impose a major constraint over bulk flow rates of resources translocated through the source-path-sink system; (3) Hydraulic conductances of plasmodesmata, linking sieve elements with surrounding phloem parenchyma cells, are sufficient to support and also regulate bulk flow rates exiting from sieve elements of release phloem. The review identifies strong circumstantial evidence that resource transport through the source-path-sink system is consistent with the high-pressure manifold model of phloem transport. The analysis then moves to exploring mechanisms that may link demand for resources, by cells of meristematic and expansion/storage sinks, with plasmodesmal conductances of release phloem. The review concludes with a brief discussion of how these mechanisms may offer novel opportunities to enhance crop biomass yields.

## Introduction

The pressure flow model of phloem transport (Münch, 1930) has gained wide acceptance. The model envisages that an osmotically-generated differential in hydrostatic pressure propels a bulk flow of phloem sap through the sieve tube (ST) system linking photosynthetic sources leaves with heterotrophic sink organs. Within sieve elements (SEs) of source leaf collection phloem, sugars (sucrose, polyols or raffinose family oligosaccharides) accumulate to high concentrations (up to 1 M). This drives an osmotic uptake of water to generate relatively high hydrostatic pressures within the SEs. Conversely unloading of osmotica from SEs, located in release phloem of sinks, causes an osmotic withdrawal of water and a consequent lowering of their hydrostatic pressures. Thus, the Münch model predicts that bulk flow of phloem sap through STs is driven by differences in hydrostatic pressures located within source and sink SEs.

Experimental observations provide persuasive support for phloem sap moving through STs by bulk flow (e.g., van Bel and Hafke, 2005) driven by hydrostatic pressure heads generated by phloem loading in source leaves (e.g., Gould et al., 2005). Recently, however, aspects of the Münch pressure flow model have been questioned on structural and physiological grounds. For instance, the Hagen-Poiseuille Law (see Equation 1), describing solvent flow driven through a pipe by a hydrostatic pressure difference (Δ*P*) for a given viscosity (η), predicts that variation in flow path geometries of STs (length–*L* and, in particular, radius–*r*), will alter their hydraulic conductances (*L*_*o*_ and see Equation 2) and hence impact axial volume flow rates (*R*_*v*_) through them.

(1) $$R_{v}=\pi r^{4}\Delta P/8\eta L$$

and where

(2) $$L_{o}=\pi r^{4}/8\eta L$$

The observed absence of any impact on axial volume flux (i.e., axial velocity derived from *R*_*v*_ expressed on the internal cross-sectional area through which solvent flow is occurring) by variations in sieve pore radii (Mullendore et al., 2010) or extent of P-protein occlusion of SE lumens (Froelich et al., 2011) has led to the suggestion that yet-to-be identified phloem transport properties are being overlooked by the Münch model (Mullendore et al., 2010; Froelich et al., 2011). A similar conclusion has been drawn from the observation that magnitudes of hydrostatic pressure gradients within STs of tall trees are inadequate to account for observed rates of bulk flow through these pipelines (Turgeon, 2010 but c.f. Jensen et al., 2012).

**Figure 1:**
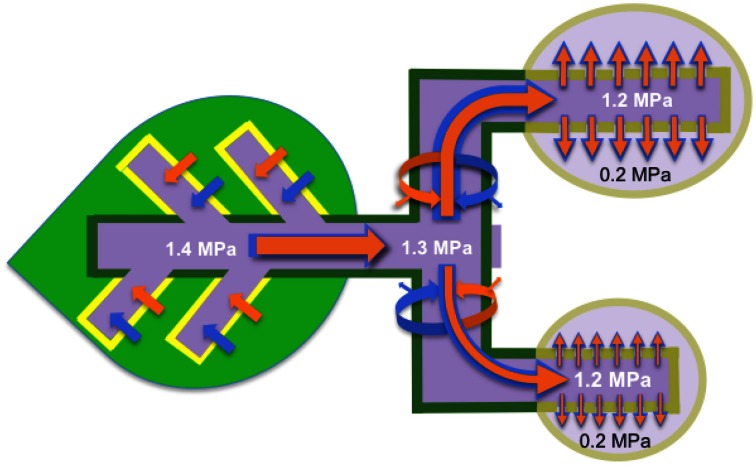
**High-pressure manifold model that describes phloem transport from source to sink and partitioning of resources (water and dissolved solutes) between sinks**. Sucrose is loaded (brown arrows) into the collection phloem (minor veins; yellow borders) of source leaves to high concentrations (dark purple) that drives an osmotic uptake of water (blue arrows). Walls of the collection phloem SEs resist the volume change with a consequent development of high hydrostatic pressures (example given, 1.4 MPa and see Table 1). STs form conduits interconnecting sources (dark green) to sinks (light purple) in a supercellular symplasm comprising collection, transport (dark green) and release (khaki) phloem. Hydrostatic pressures, generated in collection phloem SEs, are rapidly transmitted throughout the entire ST system and maintained by pressure-dependent retrieval of leaked sucrose and hence water (curved brown and blue arrows respectively). Thus STs are conceived to function as high-pressure conduits rendering resources equally available throughout a plant. Transported resources are unloaded from the release phloem SE/CC complexes as a bulk flow through high resistance (low conductance *L*_*o*_ and see Equation 2) plasmodesmal pathways into the sink cells. SE/CC unloading imposes the greatest constraint over resource flow through the source-transport-sink pathway. As a consequence, resource partitioning between sinks is finely regulated by their relative hydraulic conductances of plasmodesmata linking SE/CC complexes with the surrounding phloem parenchyma cells.

A variant of the Münch pressure flow model, the high-pressure manifold model of phloem transport (Fisher, 2000 and see Figure 1), may serve to reconcile at least some of these questions. To this end, the high-pressure manifold model of phloem transport is evaluated in relation to known properties and characteristics of phloem transport. The review commences by distilling out the key features of the high-pressure model of phloem transport followed by evaluating each of these against current knowledge of phloem transport biology.

## High-pressure manifold model of phloem transport

Key elements of the high-pressure manifold model of phloem transport articulated by Fisher (2000) and captured in Figure 1 are:

1. High hydrostatic pressures are maintained within STs throughout the length of the transport phloem with minimal decreases between collection and release phloem SEs (Figure 1). Magnitudes of these pressures are primarily generated by activities of processes determining the level to which solutes are accumulated in collection phloem SEs.
2. Large differentials in hydrostatic pressure between SEs and phloem parenchyma cells of release phloem are generated by phloem sap being expelled, by bulk flow, through paths of low hydraulic conductance (i.e., the manifold and see Equation 2) provided by plasmodesmata interconnecting these cells.
3. Phloem unloading rates are regulated by hydraulic conductances (Equation 2) of the unloading pathway rather than the axial hydrostatic pressure differential (Equation 1). In this context, the latter is comparable between a source and an array of sinks irrespective of their rates of phloem unloading (Figure 1).
4. A considerable component of the regulation of resource partitioning between sinks is mediated by the relative hydraulic conductances (Equation 2) of symplasmic unloading pathways linking SEs with phloem parenchyma cells in the release phloem (Figure 1).

Our analysis of the high-pressure manifold model of phloem transport primarily rests on evaluating the extent to which the model is consistent with known properties of resource flow through the phloem. Of necessity much of this analysis will focus on phloem transport in herbaceous plants where most experimental studies have been undertaken. However, with appropriate caveats, the analysis is extended to phloem transport in trees.

## Hydrostatic pressure gradients in sieve tubes—steep or moderate?

Bulk flow in the Münch model is conceived to be restricted to STs alone in which substantial hydrostatic pressure gradients develop from their source to sink ends. In contrast, the high-pressure manifold model (Fisher 2000 and see Figure 1) envisages the entire plant ST system is strongly pressurized with a minimal differential in hydrostatic pressures between collection and release phloem SEs. However, a major hydrostatic pressure differential occurs as a result of the substantial frictional drag imposed on an ongoing bulk flow through plasmodesmata (the manifold) interconnecting release phloem SEs with adjacent parenchyma cells (Figure 1). Literature reporting measures of ST pressures to test the proposition that hydrostatic pressure gradients between source and sink ends of ST systems are moderate is reviewed below.

Early attempts to directly measure hydrostatic pressures of STs [e.g., by manometry or pressure transducers using the phloem pressure probe described by Hammel (1968)] or indirectly (as the difference between ST sap water and solute potentials) were considered to be compromised respectively by technical short-comings (e.g., an incomplete seal around the inserted Hammel hypodermic syringe needle, and its destruction of STs) or the assumption that STs were at water equilibrium with their surrounding apoplasms (Milburn and Kallarackal, 1989). Never-the-less, estimates of ST hydrostatic pressures, deduced from measures of ST sap osmotic potentials and xylem water potentials, closely approximate those obtained using manometry or pressure transducers (e.g., see Wright and Fisher, 1980; Sovonick-Dunford et al., 1981). These findings indicate that, under most circumstances, STs are at water potential equilibrium (see Thompson and Holbrook, 2003).

The most reliable direct measures of SE hydrostatic pressures have been obtained using a methodology, developed by Wright and Fisher (1980), consisting of micromanometers glued to severed aphid stylets inserted into conducting STs. The challenging nature of the technique accounts for the few published studies, mostly carried out using herbaceous species. Averaged hydrostatic pressure values for leaves and stems need to be interpreted with caution, as it is likely that ST hydrostatic pressures oscillate with fluctuations in xylem water potentials caused by changes in transpiration loads (Lee, 1981). Such temporal oscillations, at least in part, account for the spread of recorded hydrostatic pressures in STs (Table 1). Unfortunately, to our knowledge, there are not any reported systematic examinations of ST hydrostatic pressures along source to sink axial pathways using aphid micromanometry. However, in broad terms, published data (Table 1) suggest that, if a source to sink gradient in hydrostatic pressure exists within STs, the gradient is minimal for herbaceous plants (and also see Turgeon, 2010). This feature, coupled with high ST hydrostatic pressures of 1.0 MPa, supports the contention that phloem transport in herbaceous species is consistent with the high-pressure manifold model (Fisher, 2000 and see Figure 1). The puzzling exception to this generalization is the very large and negative differential between SE hydrostatic pressures located in peduncles and the crease vein of wheat grains (Table 1) for which there is no plausible explanation at this time (see Fisher and Cash-Clark, 2000b).

**Table 1:** **Measures of sieve tube hydrostatic pressures (MPa) using micromanometers glued to severed aphid stylets inserted into translocating sieve tubes, in specified plant organs and species**.

**Plant species**	**Organ**	**Hydrostatic pressure**	**References**
*Hordeum vulgare*	Leaf	0.8–1.4	Gould et al., 2005
(Barley)	Root	1.62 ± 0.05	Gould et al., 2004a
*Sonchus oleraceus*	Leaf	1.0–1.5	Gould et al., 2005
(Sow thistle)	Stem	0.7–1.0	Gould et al., 2004b
*Triticum aestivum*	Peduncle	2.35 ± 0.56	Fisher and Cash-Clark, 2000b
(Wheat)	Grain	1.16 ± 0.26	
*Salix babylonica*	Leaf	0.51–0.93	Wright and Fisher, 1980
(Willow)	Bark strip	0.47–1.2	Wright and Fisher, 1983

Whether ST hydrostatic pressures and their axial gradients in trees comply with the high-pressure manifold model of phloem transport is rendered problematic by the paucity of suitable data sets. The only available measures of hydrostatic pressures determined by aphid stylet micromanometry are for willow (Wright and Fisher, 1980, 1983) and these are 0.2–0.5 MPa lower than those reported for herbaceous species (Table 1). However, their lower hydrostatic pressures may arise from the experimental conditions under which these were measured (Turgeon, 2010). For willow, leaf measures of ST hydrostatic pressures were determined using potted plants equilibrated under laboratory light well below the light compensation point (Wright and Fisher, 1980) and stem measures were carried out on isolated bark strips (Wright and Fisher, 1983). Both these experimental conditions would be expected to decrease the pool size of sugars available for phloem loading causing phloem sap osmotic potentials to be elevated thus lowering ST hydrostatic pressures. An assertion supported by measures of ST hydrostatic pressures ranging from 0.5 to 1.7 MPa in stems of 15 m tall white ash (*Fraxinus americana*) trees during the photoperiod (Lee, 1981; Sovonick-Dunford et al., 1981). These values are probably underestimates of their actual ST hydrostatic pressures since they were obtained using the Hammel (1968) pressure recording system (Milburn and Kallarackal, 1989). Furthermore phloem sap osmotic potentials of willow and poplar were much lower than those found in herbaceous species raised under high light conditions (Turgeon, 2010). Thus tree ST hydrostatic pressures are considered to be comparable to, or even greater than, those of herbaceous species.

Measures of ST hydrostatic pressure of willow leaf and stem by aphid stylet micromanometry (Table 1), and of white ash stems at different heights by the Hammel (1968) device (Lee, 1981), do not indicate the presence of any substantial axial gradients in ST hydrostatic pressure. However, source to sink gradients in phloem sap sugar concentrations have been detected in young (Pate and Arthur, 2000) but not well-established (Merchant et al., 2012) *Eucalyptus globulus* trees growing in plantations. Overall, the available data suggest that, at least in certain circumstances, phloem transport in trees can exhibit high ST hydrostatic pressures with minimal gradients between source and sink ends of the ST system consistent with the high-pressure manifold model (Figure 1).

Recent modeling of Münch pressure flow to optimize translocation velocity led to deriving a simple scaling relationship between ST radii with leaf (loading) and stem (transport) lengths that accounts for phloem translocation by Münch pressure flow in plant heights spanning several orders of magnitude (Jensen et al., 2011, 2012). In addition, earlier modeling predicted minimal hydrostatic pressure differentials between source and sink were essential for integrated functioning of the phloem transport system throughout the plant body (Thompson, 2006 and references cited therein). The latter model mimics the high-pressure model of phloem translocation and, of necessity, relies on ST conductances (Equation 2) scaling with plant height (see Jensen et al., 2012). However, as argued below, other features of phloem transport also may contribute to minimizing the hydrostatic pressure differential between source and sink ends of the phloem pathway.

## Conditions for high hydrostatic pressures throughout sieve tube system with minimal gradients from source to sink

In addition to highly conductive STs, high hydrostatic pressures throughout the ST system accompanied by minimal hydrostatic pressure gradients from source to sink depend upon:

### Potential capacity of phloem loading exceeds phloem unloading

Under conditions of source limitation, where phloem-unloading rates of sugars (and other phloem sap solutes) exceed the potential capacity of phloem loading, phloem sap concentrations of sugars, accompanied by ST hydrostatic pressures, decline until a new equilibrium between loading and unloading rates are reached. This condition could cause a pronounced axial gradient in ST hydrostatic pressure from source to sink to develop. The corollary is sink limitation whereby the potential capacity for phloem loading of sugars exceeds that of phloem unloading. For this condition, any increase in rates of phloem unloading is met by a commensurate increase in phloem loading. This condition would sustain high phloem sap sugar concentrations and hence ST hydrostatic pressures throughout the system.

There are no direct measures of rates of phloem loading and unloading. However, there is considerable indirect evidence suggesting that potential capacities of leaf photosynthesis arranged in series with phloem loading exceed those of phloem unloading into expanding and storage sinks. For instance, under optimal environmental conditions for leaf photosynthesis, increasing sink/source ratios by experimentally attenuating sugar export from a portion of leaves (by their removal or shading) results in elevated rates of sugar export from the untreated leaves of trees (Pinkard et al., 2011), monocots (McCormick et al., 2006) and herbaceous eudicots (Borrás et al., 2004). Similarly transgenic-mediated increases in sink capacities to store or utilize sugars, also lead to enhancing leaf photosynthesis and phloem loading (e.g., Smidansky et al., 2007; Wu and Birch, 2007). Thus under optimal conditions, plant growth tends to be sink-limited and hence phloem transport likely functions as a high pressure manifold system (see above). However, source-limitation can arise in certain circumstances including an excess of reproductive development (Qian et al., 2012), through abiotic stress (Körner, 2003) or during periods of replenishing stores of non-structural carbohydrates depleted by episodes of abiotic stress in perennial woody plants (Sala et al., 2012). Under conditions of source limitation, phloem hydrostatic pressures are predicted to be depressed and exhibit a more pronounced axial source to sink gradients in ST hydrostatic pressures. Thus, the influence of a high-pressure manifold system would be diminished under source limitation and hydraulic conductances (Equation 2) of STs could assume an increasing influence.

### Axial hydraulic conductances of sieve tubes are considerably higher than those of phloem unloading pathways

If exit of phloem sap from SEs at sinks dominates regulation of axial flow through STs from source to sink, it follows that any axial gradient in ST hydrostatic pressure would be minimized consistent with the high-pressure manifold model of phloem transport (Figure 1 and see Point 1). That ST structure, and particularly sieve plates, do not exert much influence over axial transport through them has been demonstrated by very elegant structural studies to obtain estimates of ST hydraulic conductivities [hydraulic conductance (see Equation 2) expressed on cross-sectional area basis through which bulk flow occurs] for a variety of plant species with differing sieve pore dimensions (Mullendore et al., 2010). The Hagen-Poiseuille Law (see Equation 1) predicts that, if ST conductivities were regulating axial transport, phloem transport velocities (volume flux—*R*_*v*_ of Equation (1) expressed on a cross-sectional area basis i.e., π*r*^2^) would vary directly with sieve pore radius raised to the second power. However, across a cohort of test species, estimates of phloem transport velocities were found to exhibit an inverse rather than the predicted direct relationship with their estimated ST hydraulic conductivities (Mullendore et al., 2010). This finding suggests that control of axial flow of phloem sap through STs is located elsewhere in the system. Similar conclusions were reached using an Arabidopsis T-DNA knock-out *seor1* mutant which had a reduced P-protein network (SEOR1 proteins) in STs (Froelich et al., 2011) Consistent with an absence of control being exercised by ST hydraulic conductances, phloem transport velocities in wild type and *seor1* mutant plants were found to be identical (Froelich et al., 2011).

More direct evidence that STs have spare transport capacity to support axial flow comes from physiological studies in which partial (up to ca 50%) surgical removal of phloem cross-sectional area in stems was found to exert little influence over axial flow rates in both woody plants (e.g., Mason and Maskell, 1928) and monocots (e.g., Wardlaw and Moncur, 1976). In contrast, large, and positive responses of axial phloem transport rates to surgical excision of release phloem are consistent with a major control over axial transport through the ST system being exercised by phloem unloading. A striking example of this phenomenon takes advantage of castor oil (*Ricinus communis*) plants whose STs do not seal upon severance, allowing phloem transport to continue. Hence rates of phloem transport can be estimated by measuring quantities (volume; mass) of phloem sap exuded from cut surfaces across specified times. The estimated transport rates can be expressed as ST fluxes based on measures of their cross-sectional areas through which axial transport takes place (Milburn and Kallarackal, 1989). In this case, excision of the apical fruit from a raceme of developing *Ricinus* fruits caused ST fluxes of sucrose through the pedicel stump to increase by 19-fold compared to pedicel ST fluxes when the fruit was attached (Kallarackal and Milburn, 1984). Fruit excision would remove any impedance imposed by phloem unloading on axial flow through STs and increase the hydrostatic pressure differential driving axial flow to the cut pedicel surface. Impedance conferred by phloem unloading cannot be estimated directly but changes in the hydrostatic pressure differential driving axial flow through pedicel STs can be estimated as follows. ST hydrostatic pressures of *Ricinus*, determined from osmotic and water potentials, approximate 1.0 MPa (Smith and Milburn, 1980a). Fruit excision would cause hydrostatic pressures of severed SEs located at surfaces of cut pedicels to drop to zero (i.e., atmospheric pressure). Lets assume that attached *Ricinus* fruits conform with the high-pressure manifold model of phloem transport whereby bulk flow continues symplasmically from release phloem SEs into adjacent sink cells with turgors in the range of 0.1–0.2 MPa (Saladié et al., 2007; Wada et al., 2009). Under these conditions, hydrostatic pressure differential driving bulk flow through the STs and ultimately through plamodesmata into fruit sink cells would be 0.9 or 0.8 MPa respectively. Thus, on reducing the sink-end hydrostatic pressure to zero, upon pedicel severance, the hydrostatic pressure differential driving axial flow through the pedicel STs would increase by 10 or 20% respectively (see Equation 1). As a consequence, the remaining 17.8- to 14.2-fold increase in the ST flux of sucrose elicited by fruit excision (Kallarackal and Milburn, 1984) must result from removing a considerable resistance to bulk flow imposed by SE unloading through a symplasmic pathway. This outcome is consistent with the high-pressure manifold model of phloem transport (Figure 1). Similar magnitudes of phloem transport rate increases were observed in wheat inflorescences when developing grains were detached and rates of phloem sap exuded from the broken pedicel surfaces determined (Fisher and Gifford, 1986).

An exemption to exit from release phloem dominating overall flow from source to sink is the influence imposed by hydraulic conductances of differentiating phloem paths linked with meristematic sinks. Root tips provide an opportunity to gain insights into the influence of the axial hydraulic conductances of differentiating symplasmic phloem paths (Stadler et al., 2005a). For example, as the *Zea mays* root meristem is approached, osmotic potentials of SE sap decline precipitously from axial steady levels of 4 MPa to 1.3–2.1 MPa (Warmbrodt, 1987). This decline suggests a marked reduction in hydraulic conductance (see Equation 2) of the axial phloem path that is linked with an increasing portion of differentiating SEs and provascular elements. Moreover, the decrease in osmotic potential along the axial path was ca 2-fold greater than the decrease in osmotic pressure from protophloem SEs to reach meristematic cells (Warmbrodt, 1987). This suggests a greater influence by the differentiating axial pathway on delivery to apical meristems rather than subsequent movement through the phloem-unloading route. These observations are consistent with phloem imported GFP-fusion proteins (ranging in molecular size from 36 kDa to 67 kDa) being restricted to protophloem SEs and not being able to enter differentiating protophloem SEs of Arabidopsis root tips (Stadler et al., 2005a).

### Turgor homeostasis of the sieve tube system

A prerequisite for the high-pressure manifold model of phloem transport (Figure 1) is for STs, extending from collection phloem in leaves through the transport phloem to release phloem in sinks, to function as cohesive units that sustain high hydrostatic pressures throughout their lengths with minimal source to sink gradients. This prerequisite depends upon satisfying the following conditions:

#### A rapid signaling system to convey local changes in solute demand throughout the entire sieve tube system

Increases in sink/source ratios, by blocking photosynthesis in all but one leaf (Fondy and Geiger, 1980), releasing phloem content through stem incisions (Smith and Milburn, 1980a) or selective warming of sink organs (Minchin et al., 2002), can cause rapid (instantaneous to within minutes) increases in rates of phloem export of recently-fixed photoassimilates from source leaves. The rapidity at which rates of phloem export from source leaves respond to altered sink/source ratios is considered to depend upon the degree of buffering of ST content by exchange from solute pools located along the transport phloem (Minchin et al., 2002 and see Figure 1). Indeed, buffering transport phloem content from these solute pools can meet altered sink demand without any need for change in export rates of recently-fixed photoassimilates (e.g., Fondy and Geiger, 1980). The effect is at its most extreme for the stem incision model whereby hydrostatic pressures in STs, upon severance, instantaneously fall to zero occasioning a decrease in their water potentials to drive an influx of water that would otherwise dilute their phloem sap concentrations (Smith and Milburn, 1980a). The change in sap hydrostatic pressure-concentration is rapidly propagated as a wave (Thompson, 2006) along the ST conduit to stimulate phloem loading (Smith and Milburn, 1980a). In this context, once phloem transport reaches a new steady-state in response to enhanced sink demand, osmotic concentrations of phloem sap remain unaltered and increased transport rates were accounted for by commensurate changes in transport velocity (Wardlaw and Moncur, 1976; Smith and Milburn, 1980a). Consistent with theory predicting the rapidity in axial propagation of pressure-concentration waves in response to localized changes in solute loading/unloading (Thompson, 2006; Mencuccini and Hölttä, 2010), remote changes in phloem transport rates, following localized heat ringing of sunflower stems, were conferred by a signal travelling at an axial velocity an order of magnitude greater than that of phloem transport (Watson, 1976 and also see Münch, 1930). Transmission of pressure-concentration waves depends upon elasticity and hydraulic conductances of STs (Thompson, 2006 and references cited therein). Whether ST conductances are capable of supporting rapid propagation of pressure-concentration waves over long-distances in tall trees is uncertain (Thompson, 2006), but considered likely (Mencuccini and Hölttä, 2010).

#### A mechanism for homeostasis of sieve tube hydrostatic pressures and osmotic solute concentrations

That pressure-concentration waves convey information to the entire ST system depends on high ST hydrostatic pressures and osmotic solute concentrations (Thompson and Holbrook, 2003) and hence concentrations of each major osmotic species, sucrose and potassium. In this context, apoplasmic phloem loading in leaves is negatively regulated by ST hydrostatic pressures in *Ricinus* (Smith and Milburn, 1980b), *Phaseolus coccinius* (Daie and Wyse, 1985), *Pisum sativum* (Estruch et al., 1989) and by an apoplasmic sucrose concentration specific mode in leaves of the halophyte, *Beta vulgaris* (Chiou and Bush, 1998). Passive loading in tall trees offsets decreasing water potentials with plant height through osmoregulatory activities of their mesophyll cells (Fu et al., 2011). To our knowledge, regulation of loading by the polymer trap mechanism in response to sink demand has not been investigated. However, at least some species, loading by the polymer trap mechanism, exhibit mixed loading and contain an element of apoplasmic loading of sucrose. Included amongst these species are herbaceous eudicots (Voitsekhovskaja et al., 2009; Gil et al., 2011). The ability of these polymer trap species to load sucrose apoplasmically provides opportunities for ST osmoregulation. Such a strategy may account for the positive relationship between decreasing leaf water potentials and considerable osmotic adjustment of phloem sap by elevated sucrose concentrations in trees of raffinose translocating *Eucalyptus globulus* growing across a rainfall gradient (Merchant et al., 2010). An inverse relationship between plasma membrane H^+^-ATPase activity localized to SE-CC complexes and ST hydrostatic pressure (Estruch et al., 1989) provides a unifying model for turgor homeostasis of STs. Thus, turgor-regulated shifts in H^+^-ATPase activity generate adjustments in the proton motive force driving proton-coupled symport of solutes into SE-CC complexes and membrane potential driving uptake of cations through membrane channels and, in particular, potassium.

The phenomenon of turgor homeostasis extends to SE-CCs of the transport phloem. This is graphically illustrated by positioning a cold block on a test stem to transiently slow axial phloem transport and recording temporal changes in ST hydrostatic pressure by aphid stylectomy/pressure transducers located up- and down-stream of the cold block. Applying this approach to sow thistle, Gould et al. (2004b) found that ST hydrostatic pressures upstream of the temperature block transiently rose (by >0.7 MPa) on stem chilling before declining, within 2 min, to values slightly above the pre-chill levels prior to the recovery of axial flow (8 min). Re-warming stems occasioned hydrostatic pressures to rapidly return to pre-chill levels. Down-stream of the cold block, ST hydrostatic pressures rapidly fell on chilling by 0.25–0.5 MPa and thereafter remained steady or slowly recovered. Irrespective, on rewarming stems, ST hydrostatic pressures rapidly returned to pre-chilled levels at sites up- and down-stream of the cold block. These responses are consistent with a rapid turgor homeostasis mediated throughout the ST system. During these hydrostatic pressure transients, sap osmotic potentials and sucrose concentrations were unaltered. These responses indicate that turgor homeostasis relied on co-ordinated radial exchange of solutes and water between STs and their surrounding apoplasm (Thorpe et al., 2005 and see Figure 1). Membrane carriers and channels facilitate solute influx down proton motive forces or membrane potentials respectively (Tegeder et al., 2012). The resulting transients in water potential differences between SEs and their surrounding apoplasms drive radial water fluxes into, and from, transport phloem SEs through aquaporins to maintain ST turgor. This phenomenon also would ensure compensation for loss in ST hydrostatic pressure due to frictional drag of the ST walls as predicted by Hagen-Poiseuille Law (see Equation 1).

## Symplasmic phloem unloading by bulk flow through a Plasmodesmal Manifold

The phenomenon of phloem unloading is not restricted to release phloem of terminal sinks such as apical meristems and developing reproductive (flowers, fruit, seeds) or storage (tubers, bulbs) organs. In particular, under high source/sink ratios, net resource flows occur from transport phloem of petioles, stems and roots into storage pools of their ground tissues in which transport phloem is embedded. A striking example of this phenomenon is accumulation of sucrose in storage parenchyma cells of sugarcane stems to concentrations that match those of the phloem sap (Patrick, 2012). Thus, consideration also will be given to those circumstances in which net resource unloading takes place from the transport phloem.

Phloem unloading may be arbitrarily divided into SE unloading and post-SE unloading which extends from phloem parenchyma cells to cellular sites of resource utilization or storage in non-vascular cells. For the purposes of evaluating the high-pressure manifold model of phloem transport, the primary focus is on SE unloading wherein resources reach the phloem parenchyma cells. However, some consideration, where relevant, will be given to post-SE unloading.

A central tenant of the high-pressure manifold model is that hydraulic conductances of the symplasmic unloading pathways, from SEs of release phloem, impose a major constraint over bulk flow of resources through the source-path-sink system (Figure 1). This distils to three key elements. First phloem sap is unloaded from SEs of release phloem through interconnecting plasmodesmata into surrounding phloem parenchyma cells and perhaps beyond. Second, phloem sap movement occurs as a bulk flow through plasmodesmata interconnecting SEs with surrounding phloem parenchyma cells. Third, hydraulic conductances of symplasmic unloading routes are significantly less than those supporting axial flow through STs from source to sink (and see Section Axial Hydraulic Conductances of Sieve Tubes are Considerably Higher than Those of Phloem Unloading Pathways).

### Phloem unloading in most sinks occurs through symplasmic routes

The most definitive experimental approach to mapping phloem-unloading pathways has been through the use of membrane-impermeant fluorochromes introduced into phloem sap as membrane-permeant esters or as tags linked to transgenically-expressed foreign molecules under the control of companion cell specific promoters.

#### Root and shoot apices

Axial delivery pathways into apical meristems comprise protophloem SEs giving way to strands of provascular cells more proximal to the apical dome. Distribution of membrane-impermeant fluorochromes, imported through the phloem, have identified putative symplasmic unloading pathways extending from terminal ends of protophloem SEs through provascular stands to reach meristematic cells in root apices of monocots (e.g., Hukin et al., 2002) and eudicots (e.g., Stadler et al., 2005a). Further back from root tips, in root elongation zones, the symplasmic domain for phloem unloading is constrained to protophloem SE-CC complexes and vascular parenchyma cells (Hukin et al., 2002; Stadler et al., 2005a). Symplasmic phloem unloading also applies to shoot apical meristems, as demonstrated by membrane-impermeant fluorochromes moving radially from protophloem SEs in sink leaves of monocots (Haupt et al., 2001) and eudicots (Stadler et al., 2005a).

#### Phloem unloading in developing seeds—sieve element unloading is symplasmic but non-sieve element routes include an obligatory apoplasmic step

Vasculatures terminate at funicular/coat boundaries of small seeds (e.g., Arabidopsis—Stadler et al., 2005b) or permeate coats of larger seeds (e.g., cereals and grain legumes—Zhang et al., 2007a). SE unloading of resources and their subsequent movement to specialized efflux cells follows symplasmic routes (Stadler et al., 2005b; Zhang et al., 2007a). Absence of symplasmic continuity between seed coat and filial (endosperm and embryo) tissues of developing seeds is bridged by specialized transport cells, located at maternal/filial interfaces, for resource efflux into, and influx from, seed apoplasmic spaces (Zhang et al., 2007a).

#### Tubers and fleshy fruits

Phloem unloading in developing potato tubers switches from an apoplasmic route in stolons to a symplasmic pathway in tubers (Viola et al., 2001). Phloem unloading pathways in certain fleshy fruits exhibit a reverse shift in phloem unloading pathway from symplasmic during their pre-storage phase to an apoplasmic one at the onset of sugar storage. Examples of this strategy include fruits of tomato (Patrick and Offler, 1996), grape (Zhang et al., 2006) and Chinese jujube –(Nie et al., 2010). In contrast, for other fleshy fruit an apoplasmic step in the phloem-unloading pathway is present throughout their development as illustrated for walnut (Wu et al., 2004), apple (Zhang et al., 2004) and cucumber (Hu et al., 2011). In all cases, phloem cell types in which phloem-imported fluorochromes are retained cannot be identified with confidence; a conclusion extending to Arabidopsis ovules (Werner et al., 2011). Based on plasma membrane surface areas and rates of unloading, phloem parenchyma cells are best placed to accommodate observed sucrose fluxes in developing tomato fruit (Offler and Horder, 1992).

#### Transport phloem

Sucrose continually escapes across plasma membranes of metaphloem SE-CC complexes by simple diffusion driven by large concentration differences between their lumens and surrounding apoplasm (Patrick, 1990 and see Figure 1). Furthermore, low plasmodesmal connectivity with adjoining phloem parenchyma cells, absence of membrane-impermeant dye movement and differing membrane potentials between the two cell types indicates metaphloem SE-CC complexes can be isolated symplasmically (e.g., Hayes et al., 1985; van Bel et al., 1994; Kempers et al., 1998). Apoplasmic unloading applies under low source/sink ratios (Patrick and Offler, 1996) where solute retrieval from the phloem apoplasm is dominated by SE-CC complexes over any other cell type by more than five-fold (Patrick and Turvey, 1981; Bieleski, 1966a,b; Deeken et al., 2002). Under high source/sink ratios, unloading from the transport phloem may switch to a symplasmic route (Grignon et al., 1989; Patrick and Offler, 1996; Stadler et al., 2005a). Symplasmic phloem unloading is a permanent feature of grass stems where barriers in anticlinal walls of bundle sheath cells block radial movement through their stem apoplasms (e.g., sugarcane—Jacobsen et al., 1992; wheat—Aoki et al., 2004; rice—Scofield et al., 2007).

### Is symplasmic sieve element unloading dominated by bulk flow?

A key element of the high-pressure manifold model of phloem transport and partitioning is that SE unloading occurs as a bulk flow of phloem sap. Direct demonstration of bulk flow through STs has proved to be a technically challenging exercise. The most compelling evidence has been real-time detection of sharp fronts of fluorochromes moving through SEs and across sieve plates by confocal laser scanning microscopy (Knoblauch and van Bel, 1998) and concurrence of phloem transport velocities of water (Windt et al., 2006), and of radiolabelled solutes (Wardlaw, 1990). Plasmodesmal diameters (40–60 nm) and lengths (400–800 nm) preclude resolving solution flows through them by current confocal microscopy or MRI technologies. In the meantime, evaluating whether bulk flow occurs through plasmodesmal canals will depend upon innovative indirect measures and observations (e.g., Liesche and Schulz, 2012).

The sub-structure of plasmodesmata supporting cell-to-cell movement of solutes and water is complex and its precise architecture is at the limit of resolution by electron microscopy. Solute and water movement is considered to occur through the so-called cytoplasmic sleeve, the space located between the centrally-located desmotubule and plasma membrane lining the plasmodesmal outer boundary. Computer assisted tomography of electron microscope images suggest that plasmodesmal cytoplasmic sleeves are divided into 2–3 nm wide microchannels delimited by protein filaments that interconnect desmotubule and plasma membrane (Fisher, 2000). These dimensions correspond with selective cell-to-cell trafficking, between various cell types of ground tissues, of membrane-impermeant fluorochromes with molecular sizes up to 800 Da (Fisher, 2000). In contrast, larger sized fluorescently-tagged molecules (e.g., dextrans, ficoll—Fisher and Cash-Clark, 2000a; GFP alone or GFP-fusion proteins—Stadler et al., 2005a,b) have been found to move from SEs to vascular parenchyma cells in root apices (up to 67 kDa—Stadler et al., 2005a), sink leaves (up to 50 kDA—Stadler et al., 2005a) and developing seeds of wheat (up to 400 kDa—Fisher and Cash-Clark, 2000a) and Arabidopsis (up to 47 kDa—Stadler et al., 2005b). In all cases, subsequent movement from vascular parenchyma to ground parenchyma cells was found to be constrained to smaller molecular sizes—root apices (up to 27 kDa—Stadler et al., 2005a); developing seeds of wheat (up to 10 kDa—Fisher and Cash-Clark, 2000a) and Arabidopsis (up to 27 kDa—Stadler et al., 2005b). Overall these findings suggest that the SE/CC complexes and vascular parenchyma cells form one symplasmic domain, distinguishable from an interconnected ground tissue symplasmic domain with lower plasmodesmal permeabilities.

Deducing the molecular dimensions of plasmodesmal microchannels accounting for these two symplasmic domains is problematical on a number of grounds. These include possible plasmodesmal damage by high molecular weight fluorochromes (Fisher and Cash-Clark, 2000a) and by pressure-induced unfolding tertiary structures of macromolecules at plasmodesmal orifices with subsequent movement of the ‘linearized’ macromolecule through the cytoplasmic sleeve (‘reptation’ and for further information, see Proseus and Boyer, 2005). With these caveats in mind, observed rapid movement of microinjected fluorochromes of known Stokes radii (see above) indicate microchannel radii of 4–8 nm for phloem symplasmic domains and 1–2 nm for non-phloem symplasmic domains. Future attempts to obtain estimates of plasmodesmal microchannel diameters could consider microinjecting conducting STs (Fisher and Cash-Clark, 2000a) with non-deformable colloidal gold particles of known Stokes radii (Proseus and Boyer, 2005).

Given the above estimates of plasmodesmal microchannel radii, the Hagen-Poiseuille Law (and see Equation 1) predicts that any symplasmic bulk flow of phloem sap exiting from SE/CC complexes will encounter considerable hydraulic resistances and hence a proportionate reduction in hydrostatic pressure upon entering recipient cells. These predictions are consistent with the presence of large (1.0 MPa) differences in hydrostatic pressures between release phloem SEs and cortical/epidermal cells of root tips measured directly using aphid stylet micromanometry and pressure probe respectively (Table 2). Similarly, large differences in cell sap osmotic potentials of SEs and adjacent cortical/epidermal cells (Table 2) reflect equally large differences in hydrostatic pressures as apoplasmic water potentials of these cells are identical in the absence any apoplasmic barriers and stable as a result of their hydraulic isolation from the remainder of the plant body (Lalonde et al., 2003). Radial bulk flow from release phloem SEs outward across various concentric layers of cell types would be expected to result in a progressive decline in cell hydrostatic pressures. This feature is apparent in estimates of cell osmotic potentials reported for maize root tips (Warmbrodt, 1987) as well as for pressure probe measurements of files of cortical cells in elongating *Ricinus* epicotyls (Meshcheryakov et al., 1992). Consistent with the high-pressure manifold model of phloem transport (Figure 1), the largest drop (ca 1.0 MPa) in osmotic and hydrostatic pressures occur between SEs and surrounding vascular parenchyma cells in root tips and developing wheat grains (Table 2).

**Table 2:** **Osmotic or hydrostatic pressures of sieve tubes and adjoining specified cells in root tips and developing wheat grains**.

**Pressure potential (MPa)**	**Root-tip cell type:**		**Pressure difference (MPa)**	**Plant species**	**References**
	**Sieve element**	**Elongating epi/cortical cell**			
Osmotic	−1.62	−0.98	−0.64	Maize	Warmbrodt, 1987
Osmotic	−1.42	−0.71	−0.71	Barley	Prichard, 1996
Turgor					
*High-K plants*	1.62	0.33	1.29	Barley	Gould et al., 2004a
*Low-K plants*	1.32	0.32	1.0		
**Pressure potential (MPa)**	**Grain cell type:**		**Pressure difference (MPa)**	**Plant species**	**References**
	**Sieve element**	**Vascular parenchyma**			
Turgor				Wheat	Fisher and Cash-Clark, 2000b
*Normal watered*	1.11	0.12	1.0		
*Water-stressed*	1.30	0.08	1.12		

Adapted from Patrick (2012).

A common, but largely untested generalization, is that the hydrodynamic radii of plasmodesmal microchannels prevent bulk flow by wall frictional drag spread throughout the breadth of the narrow water column. The data generated by Don Fisher's investigations of phloem unloading in developing wheat grains is sufficiently robust and complete (see Fisher and Cash-Clark, 2000a,b and references cited therein) to test whether plasmodesmal microchannel dimensions and densities are sufficient to support observed volume flow rates assuming that transport in these microchannels conform to the Hagen-Poiseuille Law (see Equation 1). Following the approach described by Fisher and Cash-Clark (2000a), the proposition was tested by determining whether sufficient numbers of plasmodesmata interconnecting SE/CC complexes with vascular parenchyma cells are present to support a known volume flow rate of 10 μL day^−1^ into a developing wheat grain across a set of microchannel radii (see Table 3). Briefly, volume flow rate through a single microchannel was computed (*R*_*v*_ – Equation 1) from which microchannel numbers supporting the observed volume flow rate were derived. From these data, microchannel numbers per plasmodesmata were estimated assuming that a single row of microchannels occupy 50% of the sleeve cross-section. Microchannel numbers per plasmodesma then allow determination of plasmodesmatal numbers required to support the observed volume flow rate (Table 3). Adequacy of microchannel radii were evaluated on the basis that estimated plasmodesmal numbers were equal to, or less than, observed plasmodesmal numbers interconnecting SE/CC complexes with vascular parenchyma cells—4.4 × 10^7^ per grain (Wang et al., 1995). These comparisons show that for the smallest Stokes radius of 0.5 nm, the observed pressure differential of 1.0 MPa (Fisher and Cash-Clark, 2000b) is not adequate to support the observed volume flow rate (Table 3). However, radii greater than this contribute spare capacity so that at 1 nm, hydrostatic pressure differentials could be lowered to 0.4 MPa before volume flow rates were compromised. Comparing our approach with that of Fisher and Cash-Clark (2000a) for a microchannel radius of 10 nm yielded identical findings of 10 × 10^4^ plasmodesmata (see Table 3) were required to support the observed volume flow rate driven by a hydrostatic pressure differential of 1.0 MPa. Overall, these analysis demonstrate that known numbers of plasmodesmata interconnecting SE/CC complexes with adjacent vascular parenchyma of developing wheat grains can support observed volume flow rates if their microchannel radii are 1 nm or greater. Significantly, this conclusion can be extended to root tips as the observed hydrostatic pressure differences (Table 2) are of a comparable magnitude to model based predictions required to account for symplasmic transport by bulk flow (Brete-Harte and Silk, 1994).

**Table 3:** **Impact of differing plasmodesmal (pd) microchannel radii^a^, predicted by the Hagen-Poiseuille Law (*see* Equation 1), on plasmodesmal numbers required to support the observed volume flow rate (*R*_*v*_)^b^ of phloem sap, of specified viscosity^c^, symplasmically unloaded from SE/CC complexes to vascular parenchyma cells of developing wheat grains at a transcellular hydrostatic pressure differential of 1.0 MPa across the interconnecting plasmodesmata of known length^d^**.

**Microchannel radius (nm)**	**Predicted^e^*R*_*v*_ per microchannel (× 10^4^ nm^3^ s^−1^)**	**Microchannel numbers supporting observed *R*_v_ (× 10^4^)^f^**	**Microchannel numbers per pd^g^**	**Pd numbers supporting observed *R***_v_ (× 10^3^)^h^
0.5	2.46	472,313	33	143,125
1.0	39.3	29,524	17	17,367
1.5	199	5832	11	5302
2.0	629	1845	8	2306
4.0	10,057	115	4	288
8.0	160,916	7	2	35.0
10.0	392,900	3	1	10.0

Plasmodesmal number estimates are compared with an observed value for developing wheat grains at the SE/CC complex and vascular parenchyma cell interface of 44,000 × 10^3^ per grain (Wang et al., 1995).

a Estimates of microchannel radii of plasmodesmata (for details, see Section Is Symplasmic Sieve Element Unloading Dominated by Bulk Flow?)

b Observed by volume flow rate (R_v_) of 11.6 × 10^13^ nm^3^ s^−1^ (Fisher, 1990).

c Averaged phloem sap viscosity of 2 × 10^−9^ MPa s (Mullendore et al., 2010).

d Cell wall thickness and hence plasmodesmatal length between SE/CC complexes and vascular parenchyma cells in developing wheat grains—500 nm (Fisher and Cash-Clark, 2000a).

e R_v_ predicted using the nominated data sets in the Hagen-Poiseuille Law (Equation 1).

f Microchannel numbers supporting observed R_v_ derived as the ratio of observed by volume flow^a^ to predicted flow rate per microchannel^e^.

g Microchannel numbers per plasmodesmata were estimated assuming that their equators (diameters) are positioned on a circumference of half the internal radius (i.e., 10.5 nm—Wang et al., 1995) of plasmodesmata interconnecting SE/CC complexes to vascular parenchyma cells and that they occupy 50% of this circumference.

h Plasmodesmal numbers supporting the observed R_v_ derived as the ratio of microchannel numbers supporting observed R^f^_v_ and microchannel numbers per plasmodesmata^g^.

If bulk flow is the primary transport mechanism by which resources are symplasmically unloaded from release phloem, it follows that rates of phloem unloading should respond proportionately to alterations in hydrostatic pressure differences between SEs and recipient sink cells. Consistent with this proposition is the finding that exposing root tips (Schulz, 1994; Pritchard et al., 2000) or abraded portions of mature stems, known to be unloading symplasmically (Patrick and Offler, 1996), to solutions containing non-permeating osmotica (mannitol and polyethylene glycol-8000 respectively), increased accumulation of phloem imported ^14^C-photoassimilates at the osmoticum-exposed sites. Since polyethylene glycol-8000 does not penetrate cell walls (Carpita et al., 1979), water withdrawal is restricted to the outer cortical cells. As a consequence, a presumptive radial gradient in turgor pressure (see Meshcheryakov et al., 1992) must be increased to account for enhanced bulk flow of phloem sap from SEs to cortical cells (Patrick and Offler, 1996). Linkage between cortical cell turgors and phloem unloading rates have been verified by real-time monitoring of cortical cell turgor and ^11^C-photoassimilate import prior to, during and following exposure of maize root tips to mannitol (Pritchard et al., 2005). Similarly, attenuating hydrostatic pressure differentials have been shown to retard phloem unloading rates of photoassimilates. For instance, potassium deficiency slowed phloem unloading in barley root tips and this response was linked with a depression of SE hydrostatic pressures whilst cortical cell pressures remained unaltered (Gould et al., 2004a and see Table 2). Conversely exposure of maize root tips to galactose dissipated the radial hydrostatic pressure gradient by increasing cortical cell turgors causing a proportionate decrease in photoassimilate unloading (Pritchard et al., 2004).

Since SE/CC complexes and vascular parenchyma cells share identical apoplasmic water potentials, turgor differentials arise from intracellular osmotic differentials and, as a result, concentration differences of phloem sap constituents. Hence diffusion must be a component of phloem unloading and the issue of the relative contributions of symplasmic phloem unloading by diffusion and bulk flow becomes one of degree. Diffusion rates (*R*_*i*_) of a solute *i* are described by Fick's First Law of diffusion as:

(3) $$R_{i}=DA\Delta C/\Delta x$$

where *D* is the diffusion coefficient of solute *i*; A is the cross-sectional area (*r* = π*r*^2^) of the diffusional pathway; Δ*C* is the concentration difference of solute *i* between either end of the diffusive pathway; Δ*x* is the length of the diffusive pathway.

To explore the potential roles of diffusion (Equation 3) and bulk flow (Equation 1) in SE unloading, published sucrose concentrations in SEs (Fisher and Gifford, 1986) and vascular parenchyma cells (Fisher and Wang, 1995) of developing wheat grains were used to estimate rates of diffusion and bulk flow through a range of microchannel radii examined in Table 3. The findings show that rates of diffusion and bulk flow were comparable for transport through a plasmodesmal microchannel radius of 2 nm for the wheat grain conditions (Table 4). Lesser radii increasingly favored diffusion whilst larger ones increasingly favored bulk flow. If the caveats around using intercellular movement of fluorescently-tagged macromolecules to obtain valid measures of plasmodesmal microchannel radii do not apply (see Section Is Symplasmic Sieve Element Unloading Dominated by Bulk Flow?), then this outcome fits nicely with symplasmic unloading from SEs through plasmodesmal microchannels, with radii in the range of 4–8 nm, being dominated by bulk flow (Table 4). As a corollary to this conclusion plasmodesmal microchannel radii of these dimensions may ensure phloem unloading occurs by bulk flow suggesting that this mechanism offers a selective advantage over diffusion in phloem unloading (for further discussion, see Section Does Bulk Flow Offer Advantages over Diffusion to Symplasmic Sieve Element Unloading?).

**Table 4:** **Comparison of estimated transport rates of sucrose by diffusion and by bulk flow through a specified range of plasmodesmal microchannel radii each with a length of 500 nm for reported sucrose concentrations (C) in SEs^a^ and vascular parenchyma cells^b^ of developing wheat grains**.

**Microchanne radius (nm)**	**Transport rates (× 10^−10^ nmol s^−1^) by:**			
	**Diffusion at a ΔC (mM) of:**		**Bulk flow at a C (mM) of:**	
	**200**	**400**	**450**	**600**
0.5	1.64	3.28	0.22	0.14
1.0	6.53	13.1	1.77	2.36
1.5	14.7	29.4	8.95	11.9
2.0	26.2	52.3	28.2	37.7
4.0	105	209	453	603
8.0	418	837	7,241	9,655

Rates of sucrose diffusion were estimated using Fick's First Law^c^ (Equation 3) for two trans-plasmodesmal differences in sucrose concentration (ΔC). Bulk flow rates of sucrose were estimated as the product of estimated volume flow rates (see Table 3 and Equation 1) and SE sucrose concentrations (C)^a^.

a Sucrose concentrations of SE sap collected from exuding pedicels of developing wheat grains ranged from 450 to 600 mM (Fisher and Gifford, 1986).

b Sucrose concentrations detected in frozen tissue slices of developing wheat grains containing vascular parenchyma cells ranged from 200 to 260 mM (Fisher and Wang, 1995). These concentrations are assumed to be representative of cytosolic sucrose concentrations in vascular parenchyma cells abutting SE/CC complexes. This assumption is an approximation as it respectively accepts that cytosolic and vacuolar sucrose concentrations are in equilibrium and that the sucrose concentration in all vascular parenchyma cells is identical.

c D_sucrose_ is 0.52 × 10^9^ nm^2^ s^−1^ in water at 25 °C. ΔC values are derived from differences between SE^a^ and vascular parenchyma^b^ sucrose concentrations.

Currently, there is only limited, and indirect, experimental evidence that offers a means of distinguishing between diffusion and bulk flow as mechanisms for phloem unloading. For instance, distribution of unloaded ^14^C-photoassimilates imported into coats of developing pea seeds occurs into distinct sectoral patterns (Grusak and Minchin, 1988). This is consistent with unloading by bulk flow rather than by diffusion that would result in ^14^C-photoassimilates being dispersed equally in all directions. Similarly, correspondence of relative accumulation patterns of symplasmically unloaded non-metabolizable substrates (^14^C-L-glucose and fluorescein) with that of ^14^C-sucrose along elongating pea epicotyls, independent of their differing symplasmic concentration gradients, suggests symplasmic unloading of these disparate solutes is primarily by bulk flow rather than diffusion (Gougler Schmalstig and Cosgrove, 1990).

### Does bulk flow offer advantages over diffusion to Symplasmic Sieve Element Unloading?

The most obvious answer to this question is embedded in a comparison of the Hagen-Poiseuille Law for bulk flow (Equation 1) and Ficks First Law of diffusion (Equation 3). That is, rates of these two processes are proportional to the radius of the conducting pipe raised to the fourth and second powers respectively. Thus, control of pipe radius can exert a four-fold greater control over rates of bulk flow compared to diffusion. This translates into an operational system whereby modest changes in microchannel dimensions can elicit substantial impacts on bulk flow rates of solute unloading from release phloem as illustrated in Tables 3 and 4.

In addition to optimizing control over phloem unloading (see above), bulk flow also avoids a dependence upon a very high level of co-ordination of unloading between individual solute species to avoid compromising phloem transport as outlined in the following scenario. If symplasmic unloading were to occur primarily by diffusion, then a mechanism must be present to ensure exit rates of each solute species and water from release phloem SEs match their rates of replenishment by axial transport. This is not a trivial requirement as diffusion rate of each solute moving through the same unloading pathway geometry depends not only on their concentration differences between SEs and vascular parenchyma cells but also on their individual diffusion coefficients (see Equation 3). Moreover phloem-imported water will diffuse at rates dictated by water potential differences between SE-CCs and vascular parenchyma cells. In these circumstances it would be very problematic for axial rates of each solute and water transported into, to match those of their diffusional exit from, release phloem SEs. As a consequence, regulation of ST hydrostatic pressures of release phloem could be compromised. This problem does not apply if SE unloading were to occur by bulk flow since solutes and solvent are transported at identical fluxes (i.e., velocities).

### Hydraulic conductances, not hydrostatic pressure differentials, regulate symplasmic unloading

Studies using root tips currently provide the most information on which to evaluate relative regulatory influences of plasmodesmal hydraulic conductances (i.e. the manifolds—see Figure 1 and Equation 2) and hydrostatic pressure differentials (Equation 1) on phloem unloading. Axial profiles of cortical cell turgors along roots of a number of plant species have been found to be invariant compared to a distinct axial profile (commencing at the root tip) of accelerating and de-accelerating zones of extension growth and hence solute import rates (Pritchard et al., 2000). The accelerating zone of root extension is symplasmically coupled with release phloem whilst the de-accelerating extension zone may (Stadler et al., 2005a) or may not be symplasmically-coupled (Pritchard et al., 2005) depending upon prevailing source/sink ratios (Patrick and Offler, 1996). Absence of any inverse relationship between accelerating rates of root extension (a measure of *R*_*v*_ and see Equation 1) and cortical cell turgor points to hydraulic conductances of plasmodesmata, located along the symplasmic unloading pathway, exerting a major regulatory influence over radial fluxes of water and hence solutes travelling from SEs to cortical cells. This explanation also accounts for transport behaviors in developing seeds whereby coat cell turgors were found to be invariant across a three-fold range of seed growth rates exhibited between French bean cultivars (Thomas et al., 2000).

Manipulating extension growth of, and hence resource import into, root tips can reproduce the above findings. For instance, acid-induced increases in root extension rates were not accompanied by any change in cortical cell turgors (Winch and Pritchard, 1998). Similarly, a step 20°C decrease in root tip temperature elicited an immediate slowing of photoassimilate import whilst cortical cell turgors remained unaffected (Pritchard et al., 2000). In addition, responses of resource unloading to these experimental perturbations were rapid (Winch and Pritchard, 1998; Pritchard et al., 2000) suggesting that any alterations in plasmodesmal hydraulic conductances likely result from plasmodesmal gating rather than *de novo* plasmodesmal formation.

The proposed role of plasmodesmal hydraulic conductances in regulating phloem unloading does not preclude shifts in hydrostatic pressure differentials between SEs and cortical cells exerting an influence such as demonstrated for potassium deficiency (Gould et al., 2004a) and decreasing extensibility of root tip cells following exposure to galactose (Pritchard et al., 2004). Therefore, there is a need to accommodate hydrostatic pressure differentials and plasmodesmal hydraulic conductances into a unified regulatory scheme. One possible scenario is hydrostatic pressure differentials exert a course control around which plasmodesmal hydraulic conductance elicit fine control. This concept is supported as follows. Since hydrostatic pressure differentials between SEs and surrounding cells represent more than 80% of SE hydrostatic pressures (Table 2), there is only modest scope for increasing hydrostatic pressure differentials and reductions will depend upon major decreases in SE, or increases in sink cell, hydrostatic pressures. Thus, at best, alterations in hydrostatic pressure differentials could impose course control over resource unloading. In contrast, for a given hydrostatic pressure differential, the Hagen-Poiseuille Law (see Equation 1) predicts that relatively small alteration in hydrodynamic radii of plasmodesmal microchannels will exert considerable fine control over their hydraulic conductances (radius to the fourth power) determining bulk flow or their permeabilities (radius raised to the second power—see Equation 3) for diffusion.

Control of phloem unloading by plasmodesmal hydraulic conductances could be exerted at two levels. First, plasmodesmal geometries (widths; lengths—Equation 2) and densities, formed during their development at various cellular interfaces located along phloem unloading pathways set upper limits for symplasmic hydraulic conductances. For example, inferred from Stoke's radii of the permeating molecules, these data yield conservative estimates of plasmodesmal microchannel radii for phloem and non-phloem domains that extend over a two-fold range (see Section Is Symplasmic Sieve Element Unloading Dominated by Bulk Flow?). In relative terms, these radii translate into a 16-fold range of hydraulic conductances. Second, plasmodesmal gating (open versus closed), mediated by callose deposition/hydrolysis around plasmodesmal neck regions, has been demonstrated to play a key role in regulating transmission of macromolecular signals orchestrating cell development (Burch-Smith and Zambryski, 2012). Plasmodesmal gating by callose deposition/removal also appears to apply to the transport of small molecules by the rapid responses to exposure of root tips to hydrogen peroxide (Rutschow et al., 2011). In addition, when root tips of hydroponically-grown pea seedlings were exposed to hyperosmotic conditions, plasmodesmal neck constrictions disappeared and their cytoplasmic sleeved expanded within 1 h of transfer to solutions containing 350 mM mannitol (Schulz, 1995). Significantly, these alterations in plasmodesmal canal geometries coincided with a three-fold increase in phloem unloading rates at the root tips (Schulz, 1994).

## How is sink demand communicated to regulate plasmodesmal conductances?

Conductance for symplasmic movement between two adjacent cells is a function of their averaged conductance of plasmodesmata summed over the density of plasmodesmata located in their shared wall. Plasmodesmal densities are under both positional and developmental control (Burch-Smith and Zambryski, 2012). Regulatory mechanisms controlling plasmodesmal densities are beginning to be discovered and these exert a predictable positive influence on overall symplasmic conductance (e.g., Burch-Smith and Zambryski, 2010; Xu et al., 2012). For root apices, plasmodesmal densities in transverse walls are higher than those in periclinal walls and both densities progressively decrease with distance from the vascular tissues (Zhu et al., 1998). This plasmodesmal network possibly contributes to canalizing longitudinal flow of solutes from the phloem to root apical meristems (Stadler et al., 2005a). A similar scenario likely applies to shoot apical meristems. In contrast, a distinguishing feature of symplasmic unloading pathways servicing expansion/storage sinks is that plasmodesmal densities are least at their SE-CC/phloem parenchyma cell interfaces (Patrick and Offler, 1996). The bottleneck imposed by plasmodesmal densities at the SE-CC/phloem parenchyma cell interface is consistent with it being a major control point for phloem unloading (and also see Sections Axial Hydraulic Conductances of Sieve Tubes are Considerably Higher than Those of Phloem Unloading Pathways and Symplasmic Phloem Unloading by Bulk Flow Through a Plasmodesmal Manifold). This feature focuses attention on hydrodynamic radii of plasmodesmal canals at this interface as a major influence over symplasmic conductance (Section Symplasmic Phloem Unloading by Bulk Flow Through a Plasmodesmal Manifold and Tables 3 and 4). How plasmodesmal conductances may be co-ordinated with temporal shifts in downstream sink demand can be speculated upon from an emerging understanding of how plasmodesmal conductances are regulated. This question is explored separately for meristematic and expansion/storage sinks.

**Figure 2:**
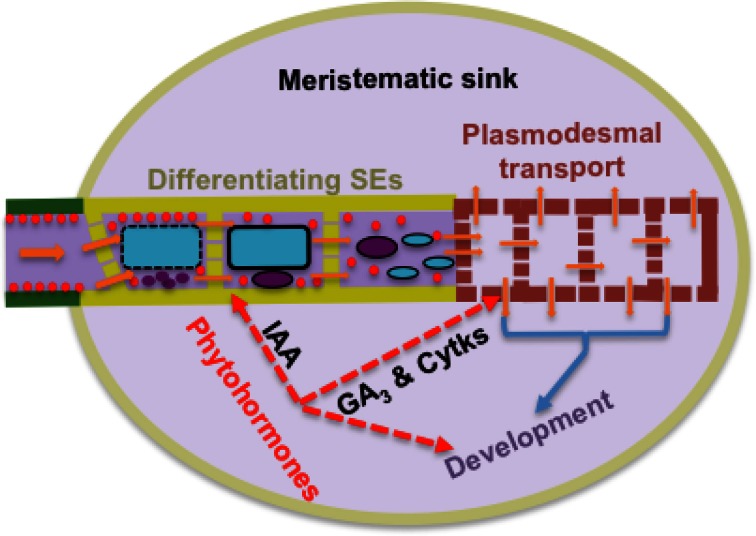
**Model of a high resistance pathway encountered by resource flow from transport phloem (green) into meristematic sinks through protophloem SEs differentiating from provascular cells (khaki) arranged in series with symplasmic movement through plasmodesmata of meristematic cells (brown)**. One tier of sink control of resource import is mediated by phytohormones integrating hydraulic conductances of this transport pathway with sink demand as illustrated for indole-3-actic acid (IAA), gibberellic acid (GA_3_) and cytokinins (Cytks). During differentiation, each developing SE forms a large central vacuole (blue) and conspicuous protein bodies and plastids (red). Their vacuole and nucleus (purple) finally degrade to leave a parietal cytoplasm of protein bodies and plastids. Co-incidentally pit fields of plasmodemata coalesce into sieve pores.

Within meristematic sinks, plant hormones regulate rates of cell division and hence sink demand. This hormone function is linked with their ability to directly act on transport processes delivering resources to hormone-enriched sites (Patrick, 1987). Fully-elongated bean stems, following surgical removal of acropetally growing regions and treating the decapitated stem stumps with plant hormones reproduces the hormonal status of meristematic sinks, but with the absence of cell division/expansion and hence altered sink demand (Patrick, 1987 and literature cited therein). Using the decapitated stem model, in which differentials in sucrose concentration or cell turgor between stem SEs and cortical cells were clamped experimentally, demonstrated that cytokinins and gibberellins stimulated phloem import of photoassimilates by increasing plasmodesmal permeabilities or conductances respectively along the symplasmic-unloading pathway at the site of hormone application (Patrick and Offler, 1996). How hormones exert their putative control of plasmodesmal permeabilities/conductances is unknown, but the rapid (h) and step change in rates of photoassimilate import by stems treated with gibberellin or cytokinin (Mulligan and Patrick, 1979; Turvey and Patrick, 1979) is consistent with post-translational regulation. Rapid (h) effects on plasmodesmal permeabilities have been reported in Arabidopsis root tips exposed to hydrogen peroxide (Rutschow et al., 2011) providing a regulatory pathway through which hormones (De Tullio et al., 2010) may or may not act on plasmodesmal conductances. At this juncture, hormonal control of plasmodesmal permeabilities/conductances awaits independent verification (cf. Burch-Smith and Zambryski, 2012 and see Rutschow et al., 2011), but offers an attractive model of matching resource delivery with resource demand by meristematic cells. Based on observations in root apices (see Section Axial Hydraulic Conductances of Sieve Tubes are Considerably Higher than Those of Phloem Unloading Pathways), the primary site at which hormonal control is likely to be exerted over bulk flow rates is on the combined hydraulic conductances of plasmodesmata and differentiating sieve pores located in the provascular strands canalizing an axial flow of resources toward the root meristem (and see Figure 2). In addition, hormonal regulation of protophloem formation from provascular strands (Aloni, 2010) is accompanied by coalescing pit fields of plasmodesmata into sieve pores with radii several orders larger than those of plasmodesmal microchannels thus conferring substantial increases in hydraulic conductances of axial transport pathways (see Equation 2).

**Figure 3:**
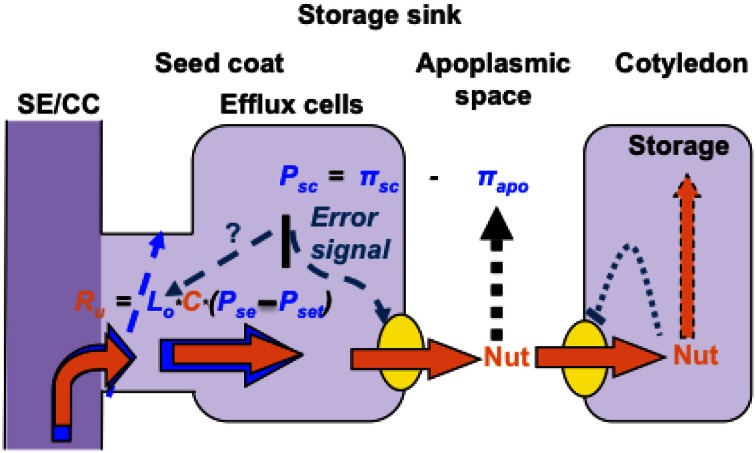
**Turgor homeostasis model of phloem unloading in developing seeds of grain legumes**. Sucrose uptake by, and storage, in cotyledons is coupled through a negative feedback transcriptional regulation of sucrose transporter activity mediated through intracellular sucrose levels. Activities of seed coat sucrose effluxers are coordinated with cotyledon demand by a turgor-homeostat mechanism that osmotically (π) detects alterations (error signal) in apoplasmic sucrose pool sizes as a deviation from a turgor (*P)* set point. Symplasmic unloading from release phloem SE/CC complexes by bulk flow is regulated by plasmodesmal hydraulic conductances (*L*_*o*_) under control of the turgor homeostat functioning as a central control hub to regulate resource flow from SE/CC complexes to ultimate storage in cotyledons.

For expansion/storage sinks, some insights into the regulatory mechanisms integrating sink demand with plasmodesmal control of resource flow along phloem unloading pathways has been obtained from observations of developing seeds. Phloem unloading pathways in developing seeds universally contain a symplasmic discontinuity at, or proximal to, their maternal and filial interfaces at which phloem-imported resources are released to, and retrieved from, the seed apoplasmic space (Zhang et al., 2007a and see Figure 3). A hint that hydraulic conductances of plasmodesmata, forming the maternal component of the symplasmic unloading pathway (Figure 3), regulate resource flows is the finding that a pharmacological block of sucrose uptake into endosperm of attached wheat grains was not accompanied by any change in sucrose concentrations located in cells forming the phloem unloading pathway in maternal grain tissues (Fisher and Wang, 1995). The absence of continued phloem unloading into, and accumulation of sucrose by, maternal grain tissues suggests that plasmodesmata linking SEs to resource release sites in the nucellus projection (Wang et al., 1995) must have immediately gated closed upon inhibiting sucrose uptake into the endosperm. The response points to a direct link between sucrose uptake by endosperm and plasmodesmal conductances at SE-CC-vascular parenchyma interfaces. This proposed relationship accounts for the observed invariance of sucrose concentrations, located at various points along phloem-unloading pathways in developing seeds, when sucrose fluxes are altered (Fader and Koller, 1985; Thomas et al., 2000). The mechanism responsible for adjusting plasmodesmal conductances at SE-CC/phloem parenchyma cell interfaces with activities of membrane transporters responsible for resource transit to, and from, seed apoplasmic spaces is not known. However, a reasoned case can be made for cell turgor integrating these disparate transport processes based on a turgor-homeostat mechanism that co-ordinates resource release to, and uptake from, seed apoplasmic spaces (Zhang et al., 2007a and see Figure 3). Cell turgor-regulated control of plasma membrane transporter activities located in seed maternal tissues is mediated through mechano-sensitive properties of the actin cytoskeleton detecting subtle turgor-induced changes in cell volume (Zhang et al., 2007b). Since components of the actin cytoskeleton form part of the plasmodesmal canal sub-structure and actin dynamics can influence plasmodesmal permeabilities (White and Barton, 2011), it then follows that plasmodesmal conductances at the SE-CC/phloem parenchyma cell interface may be co-ordinately regulated with activities of membrane transporters to meet alterations in resource demand by filial tissues of developing seeds. In addition, actin-dependent control of plasmodesmal hydraulic conductances, through a turgor-homeostatic mechanism, likely operates to regulate phloem unloading of resources during the pre-storage phase of seed development. To directly test this proposed mechanism experimentally will not be a trivial exercise.

## The high-presspure manifold model and crop biomass yield potential

Crop biomass yield potential is the harvested yield per plant of any cultivar raised under optimal environmental conditions for its development in the absence of any limitation imposed by abiotic or biotic stresses (Evans and Fischer, 1999). This yield parameter has proven to be a reproducible and robust selection tool in conventional breeding programs for crop yield realized at the farm gate (Fischer and Edmeades, 2010). Components of crop biomass yield are the number of harvested units (seed, fruit, tubers etc.) per plant and the average biomass per harvested unit. Numbers of harvested units per plant are determined during the meristematic (pre-storage) phase of yield organ development. For example, at fruit and seed set, their relative levels of loss through abortion largely govern their numbers at harvest. In addition, longevity of the meristematic phase determines cell numbers of each harvestable unit and hence their harvested size. Both abortion and meristematic activity are very sensitive to abiotic stresses (Ruan et al., 2012). Cessation of meristematic activity is replaced by cell expansion and accumulation of storage product(s)—the so-called storage phase of harvestable organ development during which crop biomass yield potential, set during the pre-storage phase, is realized.

The elements carbon, hydrogen and oxygen account for ca 90% of crop biomass yield. These elements reach the harvested units as phloem-imported sugars that form the major osmotica generating ST hydrostatic pressures propelling bulk flow from source to sink. The preceding analysis of the high-pressure manifold model of phloem transport identified hydraulic conductances of plasmodesmata, located at the sink-end of phloem pathways, as key regulators of bulk flow rates from source to sink and for partitioning of flow rates between competing sinks (Figure 1). This should position the high-pressure manifold model of phloem transport as a core determinant of crop biomass yield potential; a claim yet to be verified experimentally. However, as reviewed below, there are hints, based on circumstantial evidence, that hydraulic conductances of phloem unloading pathways play a central role in determining harvested organ numbers per plant as well as influencing their rates of storage product accumulation and hence their harvested biomass.

The pre-storage phase of harvestable organ development is photoassimilate limited as demonstrated by positive responses of harvestable organ numbers to elevated source/sink ratios (Ruan et al., 2012). Interpreted in terms of the high-pressure manifold model of phloem transport, increased source output of photoassimilates ultimately results in elevated sucrose concentrations accumulating in, and hence raising hydrostatic pressures of, the collection and transport phloem. This would result in increased hydrostatic pressure differentials forming across the low hydraulic conductance axial pathways of developing provascular stands linking the transport phloem with meristems of developing harvested organs (Section Conditions for High Hydrostatic Pressures Throughout Sieve Tube System With Minimal Gradients from Source to Sink). As a consequence, bulk flow rates of phloem sap increase into these tissues as shown by altering ST hydrostatic pressure potentials of release phloem and ground cells of root tips (Gould et al., 2004a; Pritchard et al., 2004). However, elevating crop photosynthesis as a yield improvement strategy at the pre-storage phase of development can be questioned on efficiency grounds since meristem carbon demand is considerably less than whole plant photoassimilate production. In addition, harvest unit abortion appears to be regulated by sugar signals averting expression of programmed cell death genes and depressing genes regulating cell division (Ruan et al., 2012). These regulatory events would require only small increases in photoassimilate import into pre-storage sinks. In this context, altering hydraulic conductances of axial pathways of developing provascular strands provides a sink specific action on resource flows and does not encounter a carbon cost. As outlined in Section How Is Sink Demand Communicated to Regulate Plasmodesmal Conductances?, plant hormones could function in this way (see Figure 2). Consistent with this proposition, transgenic upregulation of hormonal levels in harvestable units during their pre-storage phase of development has been found to result in increasing numbers of harvestable units. For example increased numbers of seeds (Radchuk et al., 2012), fruit (Bartrina et al., 2011) and tubers (Prat, 2010) per plant. For developing seeds, another layer of control could be exercised by sink demand of their filial tissues sensed through a turgor homeostatic mechanism that adjusts plasmodesmal hydraulic conductances of phloem unloading pathways delivering resources to these sinks (see Figure 3 and associated text in Section How Is Sink Demand Communicated to Regulate Plasmodesmal Conductances?). This scenario is illustrated by transgenically expressing a post-translationally de-regulated *Zea mays* ADP-glucosepyrophosphorylase, under control of an endosperm-specific promoter, in pre-storage grains of wheat (Smidansky et al., 2002), rice (Smidansky et al., 2003) and maize (Hannah et al., 2012). The transgenic plants supported 36, 19 and 23 percent increases respectively in grain numbers per plant and hence their biomass yields without compromising size (biomass) of their individual grains. Interestingly, increased sink demand of the developing seeds led to a de-repression of leaf photosynthesis in wheat (Smidansky et al., 2007) consistent with spare phototosynthetic potential during the pre-storage phase of seed development.

The storage phase filling rates are characteristically sink-limited (Borrás et al., 2004). The low hydraulic conductances of plasmodesmata interconnecting SE-CC complexes with adjacent phloem parenchyma cells in the release phloem of storage phase sinks could well account for a component of their sink limitation (see Section How Is Sink Demand Communicated to Regulate Plasmodesmal Conductances?). For example, in order to generate a 10% increase in bulk flow rates through these plasmodesmata in storage-phase wheat grain would require depressing phloem parenchyma cell hydrostatic pressure to zero (see Table 2). In contrast, the turgor-homeostat linkage between filial sink demand and plasmodesmal hydraulic conductances (Figure 3 and see Section How Is Sink Demand Communicated to Regulate Plasmodesmal Conductances?) readily could be accommodated by increasing radii of their plasmodesmal canals by 1.3% (Table 3) as predicted by the Hagen-Poiseuille Law (see Equation 1). That such an outcome is achievable has been demonstrated by transgenic increases in sucrose transporter activity of filial tissues of developing pea (Rosche et al., 2002) and wheat (Weichert et al., 2010) seeds increasing seed biomass gains by 23 and 16 percent respectively under controlled environmental conditions.

Overall, the above circumstantial evidence indicates that opportunities may well exist to improve crop biomass yield through manipulating resource transport informed by the high-pressure manifold model of phloem transport.

## Conclusions

Reviewing current knowledge of phloem transport leads to a strong circumstantial case being made for the high-pressure manifold model of phloem transport. This is considered sufficient to warrant further study of the model. Initially, most effort could be directed to increasing knowledge of the plasmodesmal sub-structures and how these influence resource flow through their plasmodesmal microchannels. In particular, it is crucial to obtain direct evidence that hydraulic conductances of plasmodesmata, linking SEs with surrounding phloem parenchyma cells, support SE unloading of phloem sap by bulk flow. This enterprise could be informed by the exciting findings emerging from investigations of water flows through nanotubes showing that, with radii of several nm, flow velocities exceed those predicted by the Hagen-Poiseuille law by four to five orders of magnitude (Majumber et al., 2005). The ensuing knowledge base should deliver novel opportunities to discover how plasmodesmal hydraulic conductances are regulated to match sink demand for resources. Incorporating these putative findings into re-working models of phloem transport (cf. Thompson and Holbrook, 2003; Jensen et al., 2011, 2012) are anticipated to make substantial contributions that will allow behaviors of phloem transport to be predicted including identifying mechanisms regulating resource partitioning. The legacy left by Don Fisher's high-pressure manifold model is ripe for productive investigation that will undoubtedly deliver substantial conceptual advances to the field of phloem transport biology and innovative approaches to increasing crop yield potential.

## Dedication

This paper is dedicated to the memory of Donald Fisher, a modest, but passionate, plant scientist. Don's contributions to advancing our conceptual knowledge of phloem transport biology were substantial, unyielding in their rigor and trail blazing in their impact on the field.

### Conflict of interest statement

The author declares that the research was conducted in the absence of any commercial or financial relationships that could be construed as a potential conflict of interest.

## References

[B1] Aloni R. (2010). The induction of vascular tissues by auxin, in Plant Hormones. Biosynthesis, Signal Transduction, Action! ed Davies P. J. (Amsterdam: Springer), 485–518 10.1007/978-1-4020-2686-7_22

[B2] Aoki N. Scofield G. N. Wang X.-D. Patrick J. W. Offler C. E. Furbank R. T. (2004). Expression and localisation of the wheat sucrose transporter TaSUT1 in vegetative tissues. Planta 219, 176–184 10.1007/s00425-004-1232-7 15014993

[B3] Bartrina I. Otto E. Strnad M. Werner T. Schmülling T. (2011). Cytokinin regulates the activity of reproductive meristems, flower organ size, ovule formation, and thus seed yield in Arabidopsis thaliana. Plant Cell 23, 69–80 10.1105/tpc.110.079079 21224426 PMC3051259

[B4] Bieleski R. L. (1966a). Accumulation of phosphate, sulphate and sucrose by excised phloem tissues. Plant Physiol. 41, 447–454 10.1104/pp.41.3.447 16656275 PMC1086364

[B5] Bieleski R. L. (1966b). Sites of accumulation in excised phloem and vascular tissues. Plant Physiol. 41, 455–466 10.1104/pp.41.3.455 16656276 PMC1086365

[B6] Borrás L. Slafer G. A. Otegui M. E. (2004). Seed dry weight response to source-sink manipulation in wheat, maize and soybean: a quantitative re-appraisal. Field Crops Res. 86, 131–146 10.1016/j.fcr.2003.08.002

[B7] Brete-Harte M. S. Silk W. K. (1994). Nonvascular, symplastic diffusion of sucrose cannot satisfy the carbon demands of growth in the primary root tip of Zea mays L. Plant Physiol. 105, 19–33 10.1104/pp.105.1.19 12232183 PMC159325

[B8] Burch-Smith T. M. Zambryski P. C. (2010). Loss of INCREASED SIZE EXCLUSION LIMIT (ISE)1 or ISE2 increases the formation of secondary plasmodesmata. Curr. Biol. 20, 989–993 10.1016/j.cub.2010.03.064 20434343 PMC2902234

[B9] Burch-Smith T. M. Zambryski P. C. (2012). Plasmodesmata paradigm shift: regulation from without versus within. Annu. Rev. Plant Biol. 63, 239–226 10.1146/annurev-arplant-042811-105453 22136566

[B10] Carpita N. Sabularse D. Montezinos D. Delmer D. P. (1979). Determination of the pore size of cell walls of living plant cells. Science 205, 1144–147 10.1126/science.205.4411.1144 17735052

[B11] Chiou T.-J. Bush D. R. (1998). Sucrose is a signal molecule in assimilate partitioning. PNAS 95, 4784–4788 9539816 10.1073/pnas.95.8.4784 PMC22568

[B12] Daie J. Wyse R. E. (1985). Evidence on the mechanism of enhanced sucrose uptake at low cell turgor in leaf discs of *Phaseolus coccineus*. Physiol. Plant 64, 547–552

[B13] Deeken R. Geiger D. Fromm J. Koroleva O. Ache P. Langenfeld-Heyser R. (2002). Loss of AKT2/3 potassium channel affects sugar loading into the phloem of Arabidopsis. Planta 216, 334–344 10.1007/s00425-002-0895-1 12447548

[B14] De Tullio M. C. Jiang K. Feldman L. J. (2010). Redox regulation of root apical meristem organization: connecting root development to its environment. Plant Physiol. Biochem. 48, 328e336 10.1016/j.plaphy.2009.11.005 20031434

[B15] Estruch J. J. Peretó J. G. Vercher Y. Beltrán J. P. (1989). Sucrose loading in isolated veins of *Pisum sativum*: regulation by abscisic acid, gibberellic acid, and cell turgor. Plant Physiol. 91, 259–265 10.1104/pp.91.1.259 16667007 PMC1061984

[B16] Evans L. T. Fischer R. A. (1999). Yield potential: it definition, measurement and significance. Crop Sci. 39, 1544–1551 10.2135/cropsci1999.3961544x

[B17] Fader G. M. Koller H. R. (1985). Seed growth rate and carbohydrate pool sizes of the soybean fruit. Plant Physiol. 79, 663–666 10.1104/pp.79.3.663 16664469 PMC1074948

[B18] Fischer R. A. Edmeades G. O. (2010). Breeding and cereal yield progress. Crop Sci. 50, S85–S98

[B19] Fisher D. B. (1990). Measurement of phloem transport rates by an indicator-dilution technique. Plant Physiol. 94, 455–462 10.1104/pp.94.2.455 16667733 PMC1077253

[B20] Fisher D. B. (2000). Long-Distance Transport, in Biochemistry and Molecular Biology of Plants, ed Buchanan B. Grissem W. Jones R. (Marylands: ASPB), 730–785

[B21] Fisher D. B. Gifford R. M. (1986). Accumulation and conversion of sugars by developing wheat grains: VI gradients along the transport pathway from the peduncle to the endosperm cavity during grain filling. Plant Physiol. 82, 1024–1030 10.1104/pp.82.4.1024 16665129 PMC1056252

[B22] Fisher D. B. Wang N. (1995). Sucrose concentration gradients along the post-phloem transport transport pathway in the maternal tissues of developing wheat grains. Plant Physiol. 109, 587–592 10.1104/pp.109.2.587 12228615 PMC157624

[B23] Fisher D. B. Cash-Clark C. E. (2000a). Sieve tube unloading and post-phloem transport of fluorescent tracers and proteins injected into sieve tubes severed aphid stylets. Plant Physiol. 123, 125–137 10.1104/pp.123.1.125 10806231 PMC58988

[B24] Fisher D. B. Cash-Clark C. E. (2000b). Gradients in water potential and turgor pressure along the translocation pathway during grain filling in normally watered and water-stressed wheat plants. Plant Physiol. 123, 139–147 10.1104/pp.123.1.139 10806232 PMC58989

[B25] Fondy B. R. Geiger D. R. (1980). Effect of rapid changes in source-sink ratio on export and distribution of products of photosynthesis in leaves of *Beta vulgaris* L. and *Phaseolus vulgaris* L. Plant Physiol. 66, 945–949 10.1104/pp.66.5.945 16661558 PMC440758

[B26] Froelich D. R. Mullendore D. L. Jensen K. H. Ross-Elliot T. J. Anstead J. A. Thompson G. A. (2011). Phloem ultrastructure and pressure flow: Sieve-Element-Occlusion-Related agglomerates do not affect translocation. Plant Cell 23, 4428–4445 10.1105/tpc.111.093179 22198148 PMC3269875

[B27] Fu Q. Cheng L. Guo Y. Turgeon R. (2011). Phloem loading strategies and water relations in trees and herbaceous plants. Plant Physiol. 157, 1518–1527 10.1104/pp.111.184820 21873572 PMC3252136

[B28] Gil L. Yaron I. Shaliton D. Sauer N. Turgeon R. Wolf S. (2011). Sucrose transport plays a role in phloem loading in CMV-infected melon plants that are defined as symplasmic loaders. Plant J. 66, 366–374 10.1111/j.1365-313X.2011.04498.x 21241389

[B29] Gougler Schmalstig J. Cosgrove D. J. (1990). Coupling of solute transport and cell expansion in pea stems. Plant Physiol. 94, 1625–1633 10.1104/pp.94.4.1625 11537472 PMC1077430

[B30] Gould N. Thorpe M. R. Minchin P. E. H. Pritchard J. White P. J. (2004a). Solute is imported to elongating root cells of barley as a pressure driven-flow of solution. Funct. Plant Biol. 31, 391–397 10.1071/FP03231 32688909

[B31] Gould N. Minchin P. E. H. Thorpe M. R. (2004b). Direct measurements of sieve element hydrostatic pressure reveal strong regulation after pathway blockage. Funct. Plant Biol. 31, 987–993 10.1071/FP04058 32688967

[B32] Gould N. Thorpe M. R. Koroleva O. Minchin P. E. H. (2005). Phloem hydrostatic pressure relates to solute loading rate: a direct test of the Münch hypothesis. Funct. Plant Biol. 32, 1019–1026 10.1071/FP05036 32689197

[B33] Grignon N. Touraine B. Durand M. (1989). 5carboxyfluoroscein as a tracer of phloem translocation. Am. J. Bot. 76, 871–877

[B34] Grusak M. A. Minchin P. E. H. (1988). Seed coat unloading in *Pisum sativum*—osmotic effects in attached versus excised empty ovules. J. Exp. Bot. 39, 543–559 10.1093/jxb/39.5.543

[B35] Hammel H. T. (1968). Measurement of turgor pressure and its gradients in the phloem of oak. Plant Physiol. 43, 1042–1048 10.1104/pp.43.7.1042 16656880 PMC1086970

[B36] Hannah L. C. Futch B. Bing J. Shaw J. R. Boehlein S. Stewart J. D. (2012). A shrunken-2 transgene increases maize yield by acting in maternal tissues to increase the frequency of seed development. Plant Cell 24, 2352–2356 10.1105/tpc.112.100602 22751213 PMC3406911

[B37] Haupt S. Duncan G. H. Holzberg S. Oparka K. J. (2001). Evidence for symplastic phloem unloading in sink leaves of barley. Plant Physiol. 125, 209–218 10.1104/pp.125.1.209 11154330 PMC61003

[B38] Hayes P. M. Offler C. E. Patrick J. W. (1985). Cellular structures, plasma membrane surface areas and plasmodesmatal frequencies of the stem of *Phaseolus vulgaris* L. in relation to radial photosynthate transfer. Ann. Bot. 56, 125–138

[B39] Hu L. Sun H. Li R. Zhang L. Wang S. Sui X. (2011). Phloem unloading follows an extensive apoplasmic pathway in cucumber (*Cucumis sativa* L.) fruit from anthesis to marketable maturing stage. Plant Cell Environ. 40, 743–748 10.1111/j.1365-3040.2011.02380.x 21707653

[B40] Hukin D. Doering-Saad C. Thomas C. R. Pritchard J. (2002). Sensitivity of cell hydraulic conductivity to mercury is coincident with symplasmic isolation and expression of plasmalemma aquaporin genes in growing maize roots. Planta 215, 1047–1056 10.1007/s00425-002-0841-2 12355166

[B41] Jacobsen K. R. Fisher D. G. Maretzki A. Moore P. H. (1992). Developmental changes in the anatomy of the sugarcane stem in relation to phloem unloading and sucrose storage. Bot. Acta. 105, 70–80

[B42] Jensen K. H. Lee J. Bohr T. Bruus H. Holbrook N. M. Zwieniecki M. A. (2011). Optimality of the Münch mechanism for the translocation of sugars in plants. J. R. Soc. Interface 8, 1155–1165 10.1098/rsif.2010.0578 21245117 PMC3119876

[B43] Jensen K. H. Liesche J. Bohr T. Schulz A. (2012). Universality of phloem transport in seed plants. Plant Cell Environ. 35, 1065–1076 10.1111/j.1365-3040.2011.02472.x 22150791

[B44] Kallarackal J. Milburn J. A. (1984). Specific mass transfer and sink-controlled phloem translocation in castor bean. Aust. J. Plant Physiol. 10, 561–568

[B45] Kempers R. Ammerlaan A. van Bel A. J. E. (1998). Symplasmic constriction and ultrastructural features of the sieve element/companion cell complex in the transport phloem of apoplasmically and symplasmically phloem-loading species. Plant Physiol. 116, 271–278 10.1104/pp.116.1.271

[B46] Knoblauch M. van Bel A. J. E. (1998). Sieve tubes in action. Plant Cell 10, 35–50 10.1105/tpc.10.1.35

[B48] Körner C. (2003). Carbon limitation in trees. J. Ecol. 91, 4–17 10.1046/j.1365-2745.2003.00742.x

[B49] Lalonde S. Tegeder M. Throne-Holst M. Frommer W. B. Patrick J. W. (2003). Phloem loading and unloading of amino acids and sugars. Plant Cell Environ. 26, 37–56 10.1046/j.1365-3040.2003.00847.x16667512

[B50] Lee D. R. (1981). Synchronous pressure-potential changes in the phloem of *Fraxinus americana* L. Planta 151, 304–308 10.1007/BF00393282 24301970

[B51] Liesche J. Schulz A. (2012). Quantification of plant cell coupling with three-dimensional photoactivation microscopy. J. Microsc. 247, 2–9 10.1111/j.1365-2818.2011.03584.x 22171617

[B52] Majumber M. Chopra N. Andrews R. Hinds B. J. (2005). Enhanced flow in carbon tubes. Nature 438, 44 10.1038/43844a 16267546

[B53] Mason T. G. Maskell E. J. (1928). Studies on the transport of carbohydrates in the cotton plant. II. The factors determining the rate and direction of movement of sugars. Ann. Bot. 42, 571–636

[B54] McCormick A. J. Cramer M. D. Watt D. A. (2006). Sink strength regulates photosynthesis in sugarcane. New Phytol. 171, 759–770 10.1111/j.1469-8137.2006.01785.x 16918547

[B55] Mencuccini M. Hölttä T. (2010). The significance of phloem transport for the speed with which canopy photosynthesis and belowground respiration are linked. New Phytol. 185, 189–203 10.1111/j.1469-8137.2009.03050.x 19825019

[B56] Merchant A. Peuke A. D. Keital C. MacFarlane C. Warren C. R. Adams M. A. (2010). Phloem sap and leaf d13C, carbohydrates, and amino acid concentrations in Eucalyptus globulus change systematically according to flooding and water deficit treatment. J. Exp. Bot. 61, 1758–1793 10.1093/jxb/erq045 20211969 PMC2852667

[B57] Merchant A. Buckley T. N. Pfautsch S. Turnbull T. L. Samsa G. A. Adams M. A. (2012). Site-specific responses to short-term environmental variation are reflected in leaf and phloem-sap carbon isotopic abundance of field grown Eucalyptus globulus. Physiol. Plant 146, 448–459 10.1111/j.1399-3054.2012.01638.x 22568657

[B58] Meshcheryakov A. Steudle E. Komor E. (1992). Gradients of turgor, osmotic pressure, and water potential in the cortex of the hypocotyl of growing Ricinus seedlings. Plant Physiol. 98, 840–852 10.1104/pp.98.3.840 16668755 PMC1080278

[B59] Milburn J. A. Kallarackal J. (1989). Physiological aspects of phloem translocation, in Transport of Photoassimilates, ed Baker D. A. Milburn J. A. (New York, NY: Lonhman Scientific and Technical Essex), 262–305

[B60] Minchin P. E. H. Thorpe M. R. Farrar J. F. Koroleva O. A. (2002). Source-sink coupling in young barley plants and control of phloem loading. J. Exp. Bot. 53, 1671–1676 10.1093/jxb/erf003 12096106

[B61] Mullendore D. L. Windt C. W. Van As H. Knoblauch M. (2010). Sieve tube geometry in relation to phloem flow. Plant Cell 22, 579–593 10.1105/tpc.109.070094 20354199 PMC2861446

[B62] Mulligan D. R. Patrick J. W. (1979). Gibberellic-acid-promoted transport of assimilates in stems of *Phaseolus vulgaris* L. Localised versus remote site(s) of action. Planta 145, 233–238 10.1007/BF00454446 24317728

[B63] Münch E. (1930). Material Flow in Plants. Translated 2003 by Milburn J. A. Kreeb K. H. Germany: University of Bremen; Jena Germany: Gustav Fischer Verlag

[B64] Nie P. Wang X. Hu L. Zhang H. Zhang J. Zhang Z. (2010). The predominance of the apoplasmic phloem-unloading pathway is interrupted by a symplasmic pathway during Chinese jujube fruit development. Plant Cell Physiol. 51, 1007–1018 10.1093/pcp/pcq054 20400534

[B65] Offler C. E. Horder B. (1992). The cellular pathway of short distance transfer of photosynthates in developing tomato fruit. Plant Physiol. 99S, 41.

[B66] Pate J. S. Arthur D. J. (2000). Uptake, partitioning and utilization of carbon and nitrogen in the phloem bleeding tree, Tasmanian blue gum (*Eucalyptus globulus*). Aust. J. Plant Physiol. 27, 869–884 10.1071/PP99149

[B67] Patrick J. W. (1987). Are hormones involved in assimilate transport? in Hormone Action in Plant Development–A Critical Appraisal, eds Hoad G. V. Jackson M. B. Lenton J. R. (Butterworths), 175–187

[B68] Patrick J. W. (1990). Sieve element unloading: cellular pathway, mechanism and control. Physiol. Plant. 78, 298–308 10.1111/j.1399-3054.1990.tb02095.x

[B69] Patrick J. W. Turvey P. M. (1981). The pathway of radial transfer of photosynthate in decapitated stems of *Phaseolus vulgaris* L. Ann. Bot. 47, 611–621

[B70] Patrick J. W. (2012). Fundamentals of phloem transport physiology, in Phloem: Molecular Cell Biology, Systemic Communication, Biotic Interactions, eds Thompson G. A. van Bel A. J. E. (London: Wiley-Blackwell Publishing), 30–60

[B71] Patrick J. W. Offler C. E. (1996). Post-sieve element transport of photoassimilates in sink regions. J. Exp. Bot. 47, 1165–1178 10.1093/jxb/47.Special_Issue.1165 21245245

[B72] Pinkard E. A. Eyles A. O'Grady A. P. (2011). Are gas exchange responses to resource limitation and defoliation linked to source:sink relationships? Plant Cell Environ. 34, 16552–11665 10.1111/j.1365-3040.2011.02361.x 21707651

[B73] Prat S. (2010). Hormonal and daylength control of potato tuberization, in Plant Hormones. Biosynthesis, Signal Transduction, Action! ed. Davies P. J. (Springer), 574–596

[B73a] Prichard J. (1996). Aphid stylectomy reveals an osmotic step between sieve tube and cortical cells in barley roots. J. Exp. Bot. 47, 1519–1524 10.1093/jxb/47.10.1519

[B74] Pritchard J. Winch S. Gould N. (2000). Phloem water relations and root growth. Aust. J. Plant Physiol. 27, 539–548 10.1071/PP99175

[B75] Pritchard J. Tomos A. D. Farrar J. F. Minchin P. E. H. Gould N. Paul M. J. (2004). Turgor, solute import and growth in maize roots treated with galactose. Funct. Plant Biol. 31, 1095–1103 10.1071/FP04082 32688977

[B76] Pritchard J. Ford-Llyod B. Newbury H. J. (2005). Roots as an integrated part of the translocation pathway, in Vascular Transport in Plants, eds Holbrook N. M. Zwieniecki M. (Amsterdam: Elsevier), 157–180

[B77] Proseus T. E. Boyer J. S. (2005). Turgor pressure moves polysaccharides into growing cell walls of *Chara corallina*. Ann. Bot. 95, 967–979 10.1093/aob/mci113 15760911 PMC4246760

[B78] Qian T. Dieleman J. A. Eling A. Marcelis L. F. M. (2012). Leaf photosynthetic and morphological responses to elevated CO_2_ concentration and altered fruit number in the semi-closed greenhouse. Sci. Hort. 145, 1–9 10.1016/j.scienta.2012.07.015

[B79] Radchuk V. Radchuk R. Pirko Y. Vankova R. Gaudinova A. Korkhovy V. (2012). A somaclonal line SE7 of finger millet (*Eleusina coracea*) exhibits modified cytokinin homeostasis and increased grain yield. J. Exp. Bot. 63, 5497–5506 10.1093/jxb/ers200 22888132 PMC3444265

[B80] Rosche E. Blackmore D. Tegeder M. Richardson T. Schroeder H. Higgins T. J. V. (2002). Seed-specific expression of a potato sucrose transporter increases sucrose uptake and growth rates of developing pea cotyledons. Plant J. 30, 165–175 10.1046/j.1365-313X.2002.01282.x 12000453

[B81] Ruan Y. L. Patrick J. W. Bouzayen M. Osario S. Fernie A. R. (2012). Molecular regulation of seed and fruit development. TIPS 17, 656–665 10.1016/j.tplants.2012.06.005 22776090

[B82] Rutschow H. L. Baskin T. I. Kramer E. M. (2011). Regulation of solute flux through plasmodesmata in the root meristem. Plant Physiol. 155, 1817–1826 10.1104/pp.110.168187 21325566 PMC3091107

[B83] Sala A. Woodruff D. R. Meinzer F. C. (2012). Carbon dynamics in trees: feast or famine? Tree Physiol. 32, 764–775 10.1093/treephys/tpr143 22302370

[B84] Saladié M. Matas A. J. Isaacson T. Jenks M. A. Goodwin S. M. Niklas K. J. (2007). A reevaluation of the key factors that influence tomato fruit softening and integrity. Plant Physiol. 144, 1012–1028 10.1104/pp.107.097477 17449643 PMC1914194

[B85] Scofield G. N. Hirose T. Aoki N. Furbank R. T. (2007). Involvement of the sucrose transporter, OsSUT1, in the long-distance pathway for assimilate transport in rice. J. Exp. Bot. 58, 3155–3169 10.1093/jxb/erm153 17728297

[B86] Schulz A. (1994). Phloem transport and differential unloading in pea seedlings after source and sink manipulations. Planta 192, 239–248 10.1007/BF01089040

[B87] Schulz A. (1995). Plasmodesmal widening accompanies the short-term increase in symplasmic phloem unloading in pea root tips under osmotic stress. Protoplasma 188, 22–37 10.1007/BF01276793

[B88] Smidansky E. D. Clancy M. Meyer F. D. Lanning S. P. Blake N. K. Talbert L. E. (2002). Enhanced ADP-glucose pyrophosphorylase activity in wheat endosperm increasaes seed yield. PNAS 99, 1724–1729 10.1073/pnas.022635299 11830676 PMC122258

[B89] Smidansky E. D. Martin J. M. Hannah C. Fischer A. M. Giroux M. J. (2003). Seed yield and plant biomass increases in rice are conferred by deregulation of endosperm ADP-glucose pyrophosphorylase. Planta 217, 656–664 10.1007/s00425-002-0897-z 12569408

[B90] Smidansky E. D. Meyer F. D. Blakeslee B. Weglaz T. E. Greene T. W. Giroux M. J. (2007). Expression of a modified ADP-glucose pyrophosphorylase large subunit in wheat seeds stimulates photosynthesis and carbon metabolism. Planta 225, 965–976 10.1007/s00425-006-0400-3 17021802

[B91] Smith J. A. C. Milburn J. A. (1980a). Osmoregulation and the control of phloem-sap composition in *Ricinus communis*. Planta 148, 28–34 10.1007/BF00385438 24311262

[B92] Smith J. A. C. Milburn J. A. (1980b). Phloem turgor and the regulation of sucrose loading in Ricinus communis. Planta 148, 42–48 10.1007/BF00385440 24311264

[B93] Sovonick-Dunford S. Lee D. R. Zimmermann M. H. (1981). Direct and indirect measurements of phloem turgor pressure in white ash. Plant Physiol. 68, 121–126 16661854 10.1104/pp.68.1.121 PMC425901

[B94] Stadler R. Wright K. M. Lauterbach C. Amon G. Gahrtz M. Feuerstein A. (2005a). Expression of GFP-fusions in Arabidopsis companion cells reveals non-specific protein trafficking into sieve elements and identifies a novel post-phloem domain in roots. Plant J. 41, 319–331 10.1111/j.1365-313X.2004.02298.x 15634207

[B95] Stadler R. Lauterbach C. Sauer N. (2005b). Cell-to-cell movement of green fluorescent protein reveals post-phloem transport in the outer integument and identifies symplastic domains in Arabidopsis seeds and embryos. Plant Physiol. 139, 701–712 10.1104/pp.105.065607 16169962 PMC1255989

[B96] Tegeder M. Ruan Y.-L. Patrick J. W. (2012). Roles of plasma membrane transporters in phloem functions, in Phloem Molecular Cell Biology, Systemic Communication, Biotic Interactions, eds Thompson G. A. van Bel A. E. J. (Hoboken: John Wiley and Sons, Inc), 63–101 10.1002/9781118382806.ch4

[B97] Thomas M. Hetherington L. Patrick J. W. (2000). Genotypic differences in seed growth rates of *Phaseolus vulgaris* L. General characteristics, seed coat factors and comparative roles of seed coats and cotyledons. Aust. J. Plant Physiol. 27, 109–118 10.1071/PP99116

[B98] Thompson M. V. (2006). Phloem: the long and the short of it. Trends Plant Sci. 11, 26–32 10.1016/j.tplants.2005.11.009 16356759

[B99] Thompson M. V. Holbrook N. M. (2003). Scaling phloem transport: water potential equilibrium and osmoregulatory flow. Plant Cell Environ. 26, 1561–1577 10.1046/j.1365-3040.2003.01080.x

[B100] Thorpe M. Minchin P. Gould N. McQueen J. (2005). The stem apoplast: a potential communication channel in plant growth regulation, in Vascular Transport in Plants, eds Holbrook N. M. Zwieniecki M. A. (Amsterdam: Elsevier Academic Press), 201–220

[B101] Turgeon R. (2010). The puzzle of phloem pressure. Plant Physiol. 154, 578–581 10.1104/pp.110.161679 20921188 PMC2949042

[B102] Turvey P. M. Patrick J. W. (1979). Kinetin-promoted transport of assimilate in stems of *Phaseolus vulgaris* L. Localised versus remote site(s) of action. Planta 147, 151–155 10.1007/BF00389516 24310971

[B103] van Bel A. J. E. van Rijen H. V. M. (1994). Microelectrode-recorded development of the symplasmic autonomy of the sieve element/companion cell complex in the stem phloem of Lupinus luteus L. Planta 192, 165–175 10.1007/BF01089031

[B104] van Bel A. J. E. Hafke J. B. (2005). Physicochemical determinants of phloem transport, in Vascular Transport in Plants, eds Holbrook N. M. Zwieniecki M. A. (Amsterdam: Elsevier Academic Press), 19–44

[B105] Viola R. Roberts A. G. Haupt S. Gazzani S. Hancock R. D. Marmiroli N. (2001). Tuberization in potato involves a switch from apoplastic to symplastic phloem unloading. Plant Cell 13, 385–398 10.1105/tpc.13.2.385 11226192 PMC102249

[B106] Voitsekhovskaja O. V. Rudashevskaya E. L. Demchenko K. N. Pakhomova M. V. Batashev D. R. Gamelei Y. V. (2009). Evidence for functional heterogeneity of sieve element-companion cell complexes in minor veins phloem of Alonsoa meridionalis. J. Exp. Bot. 60, 1873–1883 10.1093/jxb/erp074 19321649

[B107] Wada H. Matthews M. A. Shakel K. A. (2009). Seasonal pattern of apoplastic solute accumulation and loss of cell turgor during ripening of *Vitis vinifera* fruit under field conditions. J Exp. Bot. 60, 1773–1781 10.1093/jxb/erp050 19386616 PMC2671625

[B108] Wang H. L. Offler C. E. Patrick J. W. (1995). Cellular pathway of photosynthate transfer in the developing wheat grain. II. A structural analysis and histochemical studies of the transfer pathway from the crease phloem to the endosperm cavity. Plant Cell Environ. 18, 373–388 10.1111/j.1365-3040.1995.tb00373.x

[B109] Wardlaw I. F. (1990). The control of carbon partitioning in plants. New Phytol. 116, 341–381 10.1007/PL00008146 33874094

[B110] Wardlaw I. F. Moncur L. (1976). Source, sink and hormonal-control of translocatioin in wheat. Planta 128, 93–100 10.1007/BF00390309 24430683

[B111] Warmbrodt R. D. (1987). Solute concentrations in the phloem and apex of the root of Zea mays. Am. J. Bot. 74, 394–402

[B112] Watson B. T. (1976). Rapid propogation of changes in velocity of translocation along the phloem pathway of *Helianthus annuus* L. Ann. Bot. 40, 659–667

[B113] Weichert N. Saalbach I. Weichert H. Kohl S. Erban A. Kopka J. (2010). Increasing sucrose uptake capacity of wheat grains stimulates storage protein synthesis. Plant Physiol. 152, 698–710 10.1104/pp.109.150854 20018590 PMC2815873

[B114] Werner D. Gerlitz N. Stadler R. (2011). A dual switch in phloem unloading during ovule development in Arabidopsis. Protoplasma 248, 225–235 10.1007/s00709-010-0223-8 21153670

[B115] White R. G. Barton D. A. (2011). The cytoskeleton in plasmodesmata: a role in intercellular transport? J. Exp. Bot. 62, 5249–5266 10.1093/jxb/err227 21862484

[B116] Winch S. Pritchard J. (1998). Acid-induced wall loosening is confined to the accelerating region of the root growing zone. J. Exp. Bot. 50, 1481–1487

[B117] Windt C. W. Vergeldt F. J. de Jager P. A. Van As H. (2006). MRI of long-distance water transport: a comparison of the phloem and xylem flow characteristics and dynamics in poplar, castor bean, tomato and tobacco. Plant Cell Environ. 29, 1715–1729 10.1111/j.1365-3040.2006.01544.x 16913861

[B118] Wright J. P. Fisher D. R. (1980). Direct measurement of sieve tube turgor pressure using severed aphid stylets. Plant Physiol. 65, 1133–1135 10.1104/pp.65.6.1133 16661346 PMC440496

[B119] Wright J. P. Fisher D. B. (1983). Estimation of the volumetric elastic modulus and membrane hydraulic conductivity of willow sieve tubes. Plant Physiol. 73, 1042–1047 10.1104/pp.73.4.1042 16663326 PMC1066604

[B120] Wu L. Birch R. G. (2007). Doubled sugar content in sugarcane plants modified to produce a sucrose isomer. Plant Biotech. 5, 109–117 10.1111/j.1467-7652.2006.00224.x 17207261

[B121] Wu G. L. Zhang X. Y. Zhang L. Y. Pan Q. H. Shen Y. Y. Zhang D. P. (2004). Phloem unloading in developing walnut fruit is symplasmic in the seed pericarp and apoplasmic in the fleshy pericarp. Plant Cell Physiol. 45, 1461–1470 10.1093/pcp/pch169 15564530

[B122] Xu M. Cho E. Burch-Smith T. M. Zambryski P. C. (2012). Plasmodesmata formation and cell-to-cell transport are reduced in decreased size exclusion limit 1 during embryogenesis in Arabidopsis. PNAS 109, 5098–5103 10.1073/pnas.1202919109 22411811 PMC3324027

[B123] Zhang L.-Y. Peng Y. B. Pelleschi-Travier S. Fan Y. Lu Y. F. Lu Y. M. (2004). Evidence for apoplasmic phloem unloading in developing apple fruit. Plant Physiol. 135, 1–13 10.1104/pp.103.036632 15122035 PMC429418

[B124] Zhang X.-Y. Wang X.-L. Wang X.-F. Xia G.-H. Pan Q.-H. Fan R.-C. (2006). A shift of phloem unloading from symplasmic to apoplasmic pathway is involved in developmental onset of ripening in grape berry. Plant Physiol. 142, 220–232 10.1104/pp.106.081430 16861573 PMC1557625

[B125] Zhang W.-H. Zhou Y. Dibley K. E. Tyerman S. D. Furbank R. T. Patrick J. W. (2007a). Nutrient loading of developing seeds. Funct. Plant Biol. 34, 314–331 10.1071/FP06271 32689358

[B126] Zhang W.-H. Patrick J. W. Tyerman S. D. (2007b). Actin filaments modulate hypoosmotic-responsive K+ efflux channels in specialised cells of developing bean seed coats. Funct. Plant Biol. 34, 874–884 10.1071/FP07138 32689416

[B127] Zhu T. Lucas W. J. Rost T. L. (1998). Directional cell-to-cell communication in the Arabidopsis root apical meristem. I. An ultrastructural and functional analysis. Protoplasma 203, 35–47 10.1007/BF01280585

